# DNA methylation in peripheral tissues and left-handedness

**DOI:** 10.1038/s41598-022-08998-0

**Published:** 2022-04-04

**Authors:** Veronika V. Odintsova, Matthew Suderman, Fiona A. Hagenbeek, Doretta Caramaschi, Jouke-Jan Hottenga, René Pool, BIOS Consortium, Bastiaan T. Heijmans, Peter A. C. ’t Hoen, Joyce van Meurs, Aaron Isaacs, Rick Jansen, Lude Franke, Dorret I. Boomsma, René Pool, Jenny van Dongen, Jouke J. Hottenga, Marleen M. J. van Greevenbroek, Coen D. A. Stehouwer, Carla J. H. van der Kallen, Casper G. Schalkwijk, Cisca Wijmenga, Lude Franke, Sasha Zhernakova, Ettje F. Tigchelaar, P. Eline Slagboom, Marian Beekman, Joris Deelen, Diana van Heemst, Jan H. Veldink, Leonard H. Van den Berg, Cornelia M. van Duijn, Bert A. Hofman, Aaron Isaacs, André G. Uitterlinden, Joyce van Meurs, P. Mila Jhamai, Michael Verbiest, H. Eka D. Suchiman, Marijn Verkerk, Ruud van der Breggen, Jeroen van Rooij, Nico Lakenberg, Hailiang Mei, Maarten van Iterson, Michiel van Galen, Jan Bot, Dasha V. Zhernakova, Rick Jansen, Peter van ’t Hof, Patrick Deelen, Irene Nooren, Peter A. C. ’t Hoen, Bastiaan T. Heijmans, Matthijs Moed, Lude Franke, Martijn Vermaat, Dasha V. Zhernakova, René Luijk, Marc Jan Bonder, Maarten van Iterson, Patrick Deelen, Freerk van Dijk, Michiel van Galen, Wibowo Arindrarto, Szymon M. Kielbasa, Morris A. Swertz, Erik. W. van Zwet, Rick Jansen, Peter-Bram ’t Hoen, Bastiaan T. Heijmans, Conor V. Dolan, Lannie Ligthart, Catharina E. M. van Beijsterveldt, Gonneke Willemsen, Eco J. C. de Geus, Jeffrey J. Beck, Erik A. Ehli, Gabriel Cuellar-Partida, David M. Evans, Sarah E. Medland, Caroline L. Relton, Dorret I. Boomsma, Jenny van Dongen

**Affiliations:** 1 Department of Biological Psychology, Vrije Universiteit Amsterdam, van der Boechorststraat 7-9, 1081 BT Amsterdam, The Netherlands; 2 Amsterdam Reproduction and Development, AR&D Research Institute, Amsterdam, The Netherlands; 3 Amsterdam Public Health Research Institute, Amsterdam, The Netherlands; 4 MRC Integrative Epidemiology Unit, Population Health Sciences, Bristol Medical School, University of Bristol, Bristol, UK; 5 Avera Institute for Human Genetics, Sioux Falls, USA; 6 The University of Queensland Diamantina Institute, The University of Queensland, Woolloongabba, Australia; 7 QIMR Berghofer Medical Research Institute, Brisbane, Australia; 8 Molecular Epidemiology Section, Department of Medical Statistics and Bioinformatics, Leiden University Medical Center, Leiden, The Netherlands; 9 Department of Human Genetics, Leiden University Medical Center, Leiden, The Netherlands; 10 Department of Internal Medicine, ErasmusMC, Rotterdam, The Netherlands; 11 Department of Genetic Epidemiology, ErasmusMC, Rotterdam, The Netherlands; 12 Department of Psychiatry, VU University Medical Center, Neuroscience Campus Amsterdam, Amsterdam, The Netherlands; 13 Department of Genetics, University Medical Centre Groningen, University of Groningen, Groningen, The Netherlands; 14 Department of Internal Medicine and School for Cardiovascular Diseases (CARIM), Maastricht University Medical Center, Maastricht, The Netherlands; 15 Department of Gerontology and Geriatrics, Leiden University Medical Center, Leiden, The Netherlands; 16 Department of Neurology, Brain Center Rudolf Magnus, University Medical Center Utrecht, Utrecht, The Netherlands; 17 Department of Epidemiology, ErasmusMC, Rotterdam, The Netherlands; 18 Sequence Analysis Support Core, Leiden University Medical Center, Leiden, The Netherlands; 19 SURFsara, Amsterdam, The Netherlands; 20 Genomics Coordination Center, University Medical Center Groningen, University of Groningen, Groningen, The Netherlands; 21 Medical Statistics Section, Department of Medical Statistics and Bioinformatics, Leiden University Medical Center, Leiden, The Netherlands

**Keywords:** Epigenomics, Behavioural genetics

## Abstract

Handedness has low heritability and epigenetic mechanisms have been proposed as an etiological mechanism. To examine this hypothesis, we performed an epigenome-wide association study of left-handedness. In a meta-analysis of 3914 adults of whole-blood DNA methylation, we observed that CpG sites located in proximity of handedness-associated genetic variants were more strongly associated with left-handedness than other CpG sites (*P* = 0.04), but did not identify any differentially methylated positions. In longitudinal analyses of DNA methylation in peripheral blood and buccal cells from children (*N* = 1737), we observed moderately stable associations across age (correlation range [0.355–0.578]), but inconsistent across tissues (correlation range [− 0.384 to 0.318]). We conclude that DNA methylation in peripheral tissues captures little of the variance in handedness. Future investigations should consider other more targeted sources of tissue, such as the brain.

## Introduction

Handedness, defined as the preferential use of one hand over the other, is established early in life and represents a highly stable trait that is thought to be accompanied by changes in brain^1^, corticospinal tract^2^, peripheral innervation and vascularization of arm skeletal muscles^3^, arm dynamics^4^, and possibly the immune system^5^. Laterality is already observable in very early stages of development: fetuses show coordinated hand movements at 8–12 weeks post-conception with more right than left arm movements in 85% of fetuses^6–8^. In children and adults, the prevalence of left-handedness is about 10%^9^. Handedness clearly clusters in families, but its inheritance pattern is not clear and the heritability of handedness is relatively low: approximately 25% in twin studies (with 95% confidence intervals (CI) ranging from 11 to 30%^10–12^) and 11.9% (95% CI 7.2–17.7) based on autosomal Identity by Descent (IBD) information from closely related individuals in the UK Biobank^13^. Early genetic hypotheses on the development of hand preference incorporated a component of randomness^14,15^: depending on which alleles were inherited, a person would be right-handed or have an equal chance of being either left- or right-handed. This randomness has also been referred to as “developmental instability”, or “fluctuating asymmetry”, representing developmental variance unique to the person^16^. Such randomness could explain monozygotic twin discordance in handedness^15^ as reported in some twin studies^17–19^, although very early studies did not confirm zygosity by DNA testing.

Candidate genes associated with handedness and brain and spinal asymmetry include leucine rich repeat transmembrane neuronal 1 (*LRRTM1*)^20^, LIM domain only 4 (*LMO4*)^21^, neuronal differentiation 6 (*NEUROD6*)^21^, proprotein convertase subtilisin/kexin type 6 (*PCSK6*)^22,23^ and the androgen receptor gene (*AR*)^24–26^. However, genome-wide association studies (GWASs) found no support for these candidate genes^27–30^, but identified multiple novel loci. Recently, the largest genome-wide association study to date, which included more than 1,5 million right-handed and 194,000 left-handed individuals, found 41 loci associated with left-handedness^13^. Besides previously described associations of left-handedness with loci that contain microtubule-associated protein 2 (*MAP2*)^29,30^ and tubulin beta class 1 (*TUBB*)^29^, the list of genome-wide significant associations was expanded with other microtubule formation and regulation genes (*TUBB3, NDRG1, TUBB4A, TUBA1B, BUB3* and *TTC28*)^13^. Thus, multiple variants were close to genes involved in microtubule functions that form part of the cytoskeleton, and play a role in neurogenesis, axon transport^31^, and brain asymmetry^32^. The results of functional analyses suggested involvement of neurogenesis and the central nervous system and brain tissues, including hippocampus and cerebrum, in the etiology of left-handedness. The variance of handedness explained by single nucleotide polymorphisms (SNP heritability) on the liability scale was 3.45% in this meta-analysis^13^. The estimate in UK Biobank was 5.9%^13^.

Partly because of the limited success of early genetic association studies, epigenomic studies have been proposed as promising targets to identify mechanisms underlying handedness^33–35^. Epigenome-wide association studies (EWAS) perform association tests for several hundred thousand of CpGs (cytosine-phosphate-guanine nucleotide base pairing) to identify differentially methylated positions (DMPs) associated with a trait. Approaches that test associations across multiple nearby correlated CpGs to identify differentially methylated regions (DMRs)^36^, or that combine multiple CpGs into DNA methylation scores^37^ help improve power by combining the effects of multiple CpG sites and reducing the number of conducted tests. The predictive value of DNA methylation by construction of individual methylation scores has been shown for several outcomes, e.g. body mass index^38^. Epigenetic variation could be one pathway to connect the hypothesized random component of handedness, and contribute to asymmetrical gene expression in the two brain hemispheres^39^ and the spinal cord^40^. The latter was supported by a genome-wide DNA methylation analysis in the right and left part of the fetal spinal cord from six samples obtained between 8 and 12 weeks post conception that detected asymmetrically methylated CpG islands of several genes^40^.

**Figure 1:**
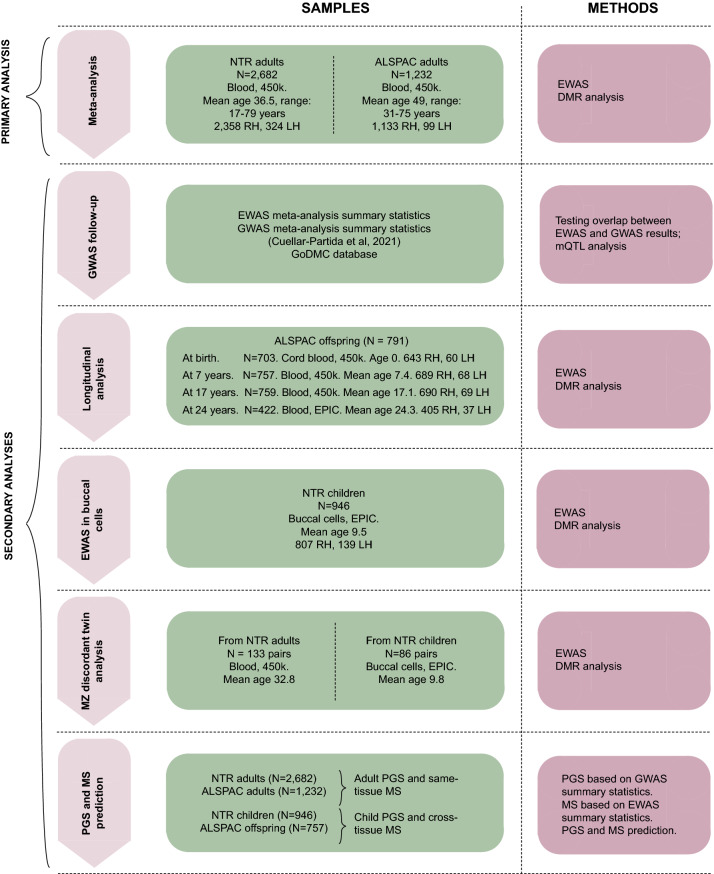
Flowchart of epigenome-wide association study of left-handedness. The flowchart summarizes the study design. The primary analyses included EWASs in NTR adults and ALSPAC adults, followed by meta-analysis to identify DNA methylation sites associated with left-handedness. The secondary analyses included: (1) left-handedness GWAS loci follow-up; (2) longitudinal analysis of DNA methylation at four ages in ALSPAC offspring; (3) analysis of buccal cell DNA methylation in NTR children; (4) analysis of DNA methylation differences between left and right-handed co-twins from NTR discordant MZ twin pairs and (5) polygenic and DNA methylation scores prediction. For left-handedness prediction, polygenic scores (PGS) were created based on left-handedness GWAS summary statistics not including NTR/ALSPAC. Methylation scores (MS) were created based on weights from EWASs in NTR adults, ALSPAC adults, NTR children and ALSPAC offspring at 7 years old to estimate the predictive performance. LH, left-handed; RH, right-handed. DMR, differentially methylated region. EWAS, epigenome-wide association study. GWAS, genome-wide association study. GoDMC, Genetics of DNA Methylation Consortium. mQTL, methylation quantitative trait locus. Blood, buccal cells indicate tissue of DNA methylation. 450 k, EPIC indicate the platform for DNA methylation measurement.

At present, no epigenome-wide association studies of handedness have been performed, and the role of DNA methylation in handedness has only been examined in small candidate-gene studies^41,42^. Here we analyzed DNA methylation data and left-handedness from two cohorts—the Netherlands Twin Register (NTR) and Avon Longitudinal Study of Parents and Children (ALSPAC). Both cohorts include methylation data in children and adults. In the ALSPAC cohort, adults and children are related (parents and offspring), and in the NTR cohort adults and children come from independent samples. We excluded ambidextrous and mixed-handed persons, and treated handedness as a dichotomous trait (left- or right-handed). First, we performed a meta-analysis of DNA methylation data from the two largest groups with DNA methylation data in adults (total sample size = 3914) to identify differentially methylated positions and differentially methylated regions associated with left-handedness. Next, we performed additional analyses in which we (1) examined if the epigenetic signal for left-handedness was enriched near previously reported GWAS loci^13^; (2) examined methylation differences between left- and right-handed twins from discordant monozygotic (MZ) twin pairs; (3) characterized the longitudinal and cross-tissue similarity of the genome-wide epigenetic signal associated with left-handedness using data from children; and (4) created methylation scores and estimated their predictive value over and above polygenic scores (Fig. 1).

## Results

### Epigenome-wide association meta-analysis of left-handedness

Tables 1, 2 and Supplementary Tables 1–4 display the characteristics of the participants included in the study. The epigenome-wide association study of left-handedness meta-analysed data from adults (*N* = 3914) with DNA methylation data in peripheral blood (Illumina, 450 k) from NTR (*N* = 2682, 34% male, mean age at methylation 36.5, standard deviation (SD) 12.7) and ALSPAC (*N* = 1232, 30% male, mean age at methylation 48.98, SD 5.55). In EWAS discovery cohorts, the prevalence of left-handedness was 12% in NTR, and 8% in ALSPAC. The prevalence of left-handedness as a function of year of birth in NTR is provided in Supplementary Table 2.

**Table 1:** Characteristics of adult cohorts included in the primary meta-analysis.

	NTR adults		ALSPAC adults	
	N = 2682		N = 1232	
	LH	RH	LH	RH
N	324 (12%)	2358 (88%)	99 (8%)	1133 (92%)
Age at blood sampling	34.3 (11.2)	36.8 (12.9)	49.1 (5.9)	49.0 (5.5)
**Sex**				
Males	119 (37%)	783 (33%)	31 (31%)	333 (29%)
Females	205 (63%)	1575 (67%)	68 (69%)	800 (71%)
Multiples	315 (97.2%)	2171 (92%)	0	0
BMI	24.2 (3.7)	24.2 (3.9)	25.80 (4.3)	26.71 (4.7)
Smoking (current)	65 (20%)	486 (21%)	37 (37%)	424 (37%)
Cell proportion	Neutrophils		B lymphocytes	
	52.8 (8.7)	52.4 (9.2)	10.8 (4.3)	10.4 (4)
	Eosinophils		CD4T	
	3.1 (1.9)	3.1 (2.3)	18.4 (7.2)	18 (6.6)
	Monocytes		CD8T	
	8.4 (2.2)	8.4 (2.4)	2.2 (3.8)	1.9 (3.1)
			Natural killer cells	
			21.9 (6.5)	20.7 (5.7)
			Granulocytes	
			45.5 (14.7)	47.9 (12.4)
			Monocytes	
			7.5 (3.4)	7.4 (3.5)

*NTR* Netherlands Twin Register, *ALSPAC* Avon Longitudinal Study of Parents and Children, *LH* left-handed, *RH* right-handed.

Whole blood DNA methylation (Illumina 450 k). Numbers in EWAS basic models are reported.

Values are presented as mean (standard deviation (SD)) or n (%).

Current smokers in ALSPAC were defined as those with cg05575921 methylation below 0.82 (see “Methods”section).

**Table 2:** Characteristics of the datasets included in secondary analyses.

	ALSPAC offspring (longitudinal)								NTR children	
	at birth N = 703		7 years old N = 757		17 years old N = 759		24 years old N = 442		N = 946	
	LH	RH	LH	RH	LH	RH	LH	RH	LH	RH
N	60 (8.5%)	643 (91.5%)	68 (9%)	689 (91%)	69 (9%)	690 (91%)	37 (8.4%)	405 (91.6%)	139(15%)	807(85%)
Age at blood sampling			7.43 (0.1)	7.4 (0.1)	16.9 (1.1)	17.1 (1.0)	24.3 (0.7)	24.3 (0.7)	9.58(1.78)	9.56(1.86)
Sex										
Males	32 (53.3%)	302 (47%)	36 (52.9%)	332 (48.2%)	36 (52.2%)	321 (46.5%)	16 (43.2%)	173 (42.7%)	71 (51%)	412 (51%)
Females	28 (46.7%)	341 (53%)	32 (47.1%)	357 (51.8%)	33 (47.8%)	369 (53.5%)	21 (56.8%)	232 (57.3%)	68 (49%)	395 (49%)
Gestational age	39.6 (1.5)	39.6 (1.4)	39.6 (1.4)	39.6 (1.6)					35.51(2.83)	35.93(2.52)
Birth weight	3567.8 (434.4)	3477.3 (493.6)	3583 (445.4)	3481 (493.3)					2369 (585.2)	2407 (533.5)
Maternal smoking during pregnancy	9 (15.2%)	74 (11.6%)	10 (14.9%)	81 (11.8%)					14(11%)	56(7%)
BMI					22.5 (3.6)	22.5 (3.6)	24.2 (3.6)	24.4 (4.5)		
Smoking (current)					20 (29.4%)	161 (23.6%)	9 (24.3%)	131 (33%)		

*NTR* Netherlands Twin Register (buccal cell DNA methylation); *ALSPAC* Avon Longitudinal Study of Parents and Children (cord blood and whole blood DNA methylation);* LH* left-handed; *RH* right-handed; *BMI* body mass index.

Numbers in EWAS basic models are reported.

Values are presented as mean (SD) or n (%).

Current smokers in ALSPAC were defined as those with cg05575921 methylation below 0.82 (see “Methods”section).

We tested 409,562 CpGs with adjustment for age, sex, smoking status, body mass index (BMI), measured or estimated cell proportions, and technical covariates. Genome-wide EWAS test statistics from each cohort separately, and from the meta-analysis, showed no inflation (Supplementary Tables 5–11). None of the associations with CpG sites reached epigenome-wide significance (i.e. Bonferroni adjusted *P* < 0.05 or false discovery rate < 5%) (Supplementary Fig. 1).The CpGs with lowest *p*-values in meta-analysis (*P* < 1 × 10^–5^) are shown in Table 3. Six of eight CpGs were located near transcription start sites on different chromosomes: in the *LRRC2* gene on chromosome 3, in the *ATP6V1B2* gene on chromosome 8, in the *CKAP4*, *GALNTR6*, and *UNC1198* genes on chromosome 12, in the *C13orf18* gene on chromosome 13, in the *MBD2* gene on chromosome 18, and in the *NTSR1* gene on chromosome 20. The average difference in DNA methylation between left-handers and right-handers at these CpGs was small (from 0.06 to 0.8%; i.e. 0.0006 to 0.008 on the methylation beta-value scale) with lower methylation level in left-handers at all CpGs except for cg13719901 (*LRRC2*) (Supplementary Figs. 2 and 3).

**Table 3:** Top differentially methylated positions from EWAS meta-analysis of left-handedness.

CpG	CHR	Position^a^	Gene	Location	*β*	SE	*P*	FDR	Direction
cg22804475	8	20,054,597	*ATP6V1B2*	TSS200	− 0.0007	0.0002	1.28 × 10^–06^	0.197	–
cg09239756	12	106,642,360	*CKAP4*	TSS1500	− 0.0032	0.0007	1.82 × 10^–06^	0.197	–
cg22541911	12	51,785,465	*GALNT6*	TSS1500	− 0.0010	0.0002	1.90 × 10^–06^	0.197	–
cg13719901	3	46,608,139	*LRRC2*	5′UTR; TSS200	0.0081	0.0017	2.60 × 10^–06^	0.197	+ +
cg02850812	13	46,961,666	*C13orf18*	TSS200	− 0.0014	0.0003	2.62 × 10^–06^	0.197	–
cg16852837	18	51,750,955	*MBD2*	1st Exon; 5′UTR	− 0.0006	0.0001	3.28 × 10^–06^	0.205	–
cg09893588	20	61,340,109	*NTSR1*	TSS200	− 0.0011	0.0003	9.12 × 10^–06^	0.256	–
cg12402132	12	121,148,554	*UNC119B*	Gene body	− 0.0009	0.0002	9.54 × 10^–06^	0.256	–

*β* is the regression coefficient for left-handedness in EWAS meta-analysis adjusted model that included N_NTR adults_ = 2663 and N_ALSPAC adults_ = 1058.

CpGs with uncorrected *P*-value < 1.0 × 10^–5^ are presented.

*CHR* chromosome; *SE* standard error; *FDR* false discovery rate; *TSS200* 200 base pairs upstream of transcription start site;*TSS1500* 1500 base pairs upstream of transcription start site; *5′UTR* 5′ untranslated region; +  +, positive direction of effect in each cohort; –, negative direction of effect in each cohort.

^a^Genome build Hg19 (build 37). See additional information on CpGs in Supplementary Table 12.

**Figure 2:**
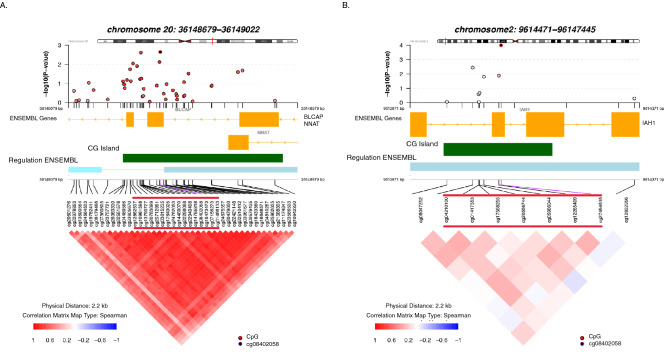
Differentially methylated regions associated with left-handedness in meta-analysis. The figure represents the differentially methylated regions at *P*-adjusted < 0.05 in meta-analysis of DMR statistics across groups of NTR adults and ALSPAC adults (*N* = 3712). The top panel of each plot depicts the EWAS *P*-values for CpGs in the differentially methylated regions (**A**) at chromosome 20; and (**B**) at chromosome 2. The x-axis indicates the position in base pair (bp) for the region, while y-axis indicates the strength of association from meta-analysis EWAS with left-handedness (adjusted model). The middle panel shows the genomic coordinates (genome build GRCh37/hg19) and the functional annotation of the region: the ENSEMBL Genes track shows the genes in the genomic region (orange); the CpG Island track shows the location of CpG islands (green); the Regulation ENSEMBL track shows regulatory regions (blue). CpGs from DMR associated with left-handedness are indicated with red lines above the correlation heatmap. More detailed information on the regions is provided in Table 4. The bottom panel shows the Spearman correlation between methylation levels of CpGs in the window. The figure was computed using the R package coMET. See additional information on CpGs from the regions in Supplementary Table 12.

The DMR meta-analysis detected two DMRs associated with left-handedness (Fig. 2, Table 4, Supplementary Table 12). The DMR on chromosome 20 (*BLCAP*; *NNAT*; 16 CpGs) had lower DNA methylation in left-handers than in right-handers (*P*-value adjusted for multiple testing (*P*_*adj*_) = 0.00004). The DMR on chromosome 2 (*IAH1*; 7 CpGs) also had lower DNA methylation in left-handers (*P*_*adj*_ = 0.03). In total, 15 of 16 CpGs in the DMR on chromosome 20 (Supplementary Figs. 4 and 5) and 6 of 7 CpGs in the DMR on chromosome 2 (Supplementary Figs. 6 and 7) were hypomethylated in left-handers. The average absolute DNA methylation difference at these regions between left-handers and right-handers based on meta-analysis regression coefficients for the individual CpGs was 0.4% for the DMR on chromosome 20, and 0.1% for the DMR on chromosome 2. Both DMRs were within CpG islands, and were not detected in the individual cohorts. Some CpG sites within left-handedness-associated DMRs have been previously associated with other traits, these associations are listed in Supplementary Table 13.

**Table 4:** Significant differentially methylated regions associated with left-handedness in meta-analysis and secondary analysis.

Cohort	Chromosome	Start	End	n CpGs	Genes	Effect size	SE	P	P_adjust_
**Meta-analysis**									
NTR and ALSPAC adults (blood DNA methylation)	chr20	36,148,679	36,149,022	16	*BLCAP*, *NNAT*	− 0.153	0.024	9.80 × 10^–11^	4.31 × 10^–05^
	chr2	9,614,471	9,614,744	7	*IAH1*	− 0.102	0.019	7.33 × 10^–08^	0.03
**Secondary analysis**									
NTR children at 9 years (buccal cell DNA methylation)	chr8	145,024,929	145,025,064	4	*PLEC1*	− 0.056	0.008	1.07 × 10^–11^	9.14 × 10^–06^
	chr22	36,011,405	36,011,843	2	*MB*	− 0.119	0.022	4.09 × 10^–08^	0.035
	chr9	111,696,674	111,697,545	10	*EELP1*, *ABITRAM*	− 0.134	0.024	4.59 × 10^–08^	0.039
	chr12	899,323	899,559	2	*WNK1*	− 0.117	0.021	4.69 × 10^–08^	0.040

Effect size is a weighted sum of the EWAS effects for each CpG site in the DMR (i.e. methylation differences between LH and RH) where the weights account for dependence between CpG sites and uncertainty in the EWAS effects (see “Methods” section). N_meta-analysis_ = 3721, N_NTR children_ = 866.

*NTR* Netherlands Twin Register. *ALSPAC* Avon Longitudinal Study of Parents and Children. *SE* standard error; *P*_adjust_ *P*-value multiplied by the total number of tests performed; the number of tests is equal to the number of regions for which DMR statistics are calculated.

### GWAS follow-up

We tested the overlap of our EWAS meta-analysis results with findings from the most recent GWAS meta-analysis of handedness^13^. CpGs located within 1 Mb window of SNPs associated with left-handedness (at *P* < 5 × 10^–8^) were on average more strongly associated with left-handedness in the EWAS meta-analysis than the other tested CpGs (*β* = 0.027, *P* = 0.04). The effect was weaker when less stringent GWAS p-value cut-offs were applied (i.e. SNPs with *P* < 1 × 10^–6^, and SNPs with *P* < 1 × 10^–5^). Importantly, in a control analysis substituting genetic loci with loci associated with type 2 diabetes^43^, we did not observe a statistically significant overlap (*β* = 0.005, *P* = 0.265) (see Supplementary Table 14, Supplementary Fig. 8).

A look-up of left-handedness associated SNPs^13^ in the methylation quantitative trait locus (mQTL) database by the Genetics of DNA Methylation Consortium (GoDMC)^44^ showed that 95% of left-handedness associated SNPs associated with methylation levels of nearby (*cis*; 86%) or distant (*trans*; 14%) CpGs (254 unique CpGs). We repeated the GWAS enrichment analysis with these CpGs driven by mQTLs removed, and obtained similar results, i.e. CpGs near GWAS loci (but not driven by significant mQTLs) were still more strongly associated with left-handedness compared to other genome-wide CpGs (*β* = 0.027, *P* = 0.027). None of the CpGs driven by mQTL was located in significant DMRs from our EWAS meta-analysis, or among the top 100 CpGs (by *p*-value) from our EWAS meta-analysis, which illustrates that our top EWAS findings are not driven by mQTL effects of the top GWAS loci.

### Longitudinal analysis

While handedness is a stable trait, DNA methylation can vary over age^45^. We analyzed DNA methylation in ALSPAC offspring measured in cord blood at birth, and in peripheral blood at 7, 17, and 24 years old (*N* = 791, Table 2 and Supplementary Table 3) to examine the association between DNA methylation and left-handedness throughout childhood and adolescence. No associations survived adjustment for multiple tests at any time point (Bonferroni adjusted *P* < 0.05; Supplementary Fig. 9e–l, Supplementary Tables 19–26). The correlations of effects for the top 100 CpG by *P*-value between time points were moderate to strong (mean correlation = 0.414; correlation range from *r* = 0.355; *P* = 0.0002 to *r* = 0.578, *P* = 1.2 × 10^–10^), except for a weak correlation between top effects at 17 years and 24 years (*r*^ALSPAC17–ALSPAC24^ = 0.079; *P* = 0.435) (Supplementary Fig. 10). There were no overlapping CpGs amongst the top 100 CpGs between analyses at different time points (Supplementary Fig. 11). Correlations between top CpG effects between ALSPAC adults (mothers and fathers) and offspring at birth were strong negative (*r*^ALSPACadults–ALSPACatbirth^ = − 0.68; *P* = 7.2 × 10^–15^) (Supplementary Fig. 12), and between ALSPAC adults and offspring at 7, 17, 24 years were weak (*r* from − 0.006 to 0.141, *P* > 0.0003) (Supplementary Fig. 10).

### DNA methylation in buccal cells

In NTR, buccal DNA methylation data (measured with the EPIC array at 787,711 CpG sites) were available in children (*N* = 946, mean age 9.5, SD = 1.85). The EWAS did not detect associations of DMPs with left-handedness (Supplementary Fig. 9m, n, Supplementary Table 27–28). The effects for top 100 CpG in EWAS of handedness in buccal cells had weak correlations with effects in blood in the meta-analysis (from *r* = 0.086, *P* = 0.39 to *r* = 0.179, *P* = 0.07), NTR adults (from *r* = 0.193, *P* = 0.05 to *r* = 0.268, *P* = 0.007), ALSPAC adults (from *r* = − 0.008, *P* = 0.94 to *r* = − 0.04, *P* = 0.95), and ALSPAC offspring at different ages (from *r* = − 0.384, *P* = 7.9 × 10^–05^ to *r* = 0.312; *P* = 0.002). Four DMRs in buccal cell DNA associated with left-handedness: DNA methylation was lower in left-handers at a DMR on chromosome 8 (4 CpGs; *P*_*adj*_ = 9.14 × 10^–06^, average difference in DNA methylation in left-handers and right-handers 0.07%), a DMR on chromosome 9 (10 CpGs; *P*_*adj*_ = 0.039, average difference 0.3%), a DMR on chromosome 12 (2 CpGs; *P*_*adj*_ = 0.04, average difference 0.98%), and a DMR on chromosome 22 (2 CpGs; *P*_*adj*_ = 0.035, average difference 1.1%) (Table 4, Supplementary Fig. 13). These DMRs did not overlap with DMRs detected in the analyses of blood methylation data. Sixteen of 18 CpGs from these regions had a lower methylation level in left-handed children than in right-handed (Supplementary Table 29).

### Sensitivity analyses

We reported the DNA methylation and left-handedness association study with adjustment for prenatal and postnatal factors that influence DNA methylation as shown in previous studies: BMI^38^ and smoking^46^ in adults, and gestational age, birth weight and prenatal maternal smoking in children^47,48^. We examined if the EWAS results for handedness differ without taking these factors into account. Across all analyses, the correlations between the effects for the top 100 CpGs were strong between the models with and without adjustment for these factors (*r* ranged from 0.99 to 1), and overlaps of the top 100 CpGs were substantial (32–87 CpGs). Adjustment for the factors increased the number of DMRs associated with left-handedness in meta-analysis (1 DMR without adjustment and 2 DMRs with adjustment), and in EWAS in children (2 DMRs without adjustment, and 4 with adjustment in buccal cells in NTR).

### Discordant MZ twins

In NTR, the DNA methylation datasets included 1039 monozygotic (MZ) adult twins with blood samples (from the meta-analysis in NTR adults) and 794 MZ children with buccal samples (from NTR children in secondary analysis) from complete twin pairs with handedness data. We found that 21% of the MZ twin adult pairs (*N* = 133 pairs) and 24% of the MZ twin child pairs (*N* = 86 pairs) were discordant for handedness. Characteristics of MZ discordant twins are presented in Supplementary Table 4. In both groups, we performed an MZ discordant within-pair EWAS analysis, comparing left- and right-handed twins. Within-pair analyses of DNA methylation of left- and right-handed twins did not identify DMPs or DMRs in blood or buccal samples at Bonferroni or FDR threshold (Supplementary Fig. 9a–d, Supplementary Tables 15–18). We compared the methylation results obtained in discordant MZ twins to the EWAS meta-analysis results for the top 100 CpGs ranked on ascending *P*-value from each analysis. To avoid sample overlap, we repeated the EWAS meta-analysis after exclusion of the MZ discordant twin pairs. Correlations between the meta-analysis top 100 effects and mean methylation differences from the within-pair analysis were weak in adults (*r*^MZ disc adults blood–Meta-analysis^ = 0.189, *P* = 0.06; *r*^Meta-analysis–MZ disc adults blood^ = 0.188, *P* = 0.06, *α* = 0.0003) and children (*r*^MZ disc children buccal–Meta-analysis^ = 0.134, *P* = 0.18; *r*^Meta-analysis–MZ disc children buccal^ = 0.252, *P* = 0.01) (Supplementary Fig. 10). There were few overlapping CpGs among the top 100 CpGs from the within-pair analyses and other analyses (Supplementary Fig. 11).

### Handedness methylation scores

To examine if variation in handedness can be predicted by DNA methylation levels across multiple CpGs, methylation scores (MS) were created. These were based on EWAS summary statistics in NTR to predict into ALSPAC, and on ALSPAC summary statistics to predict into NTR given the following p-value thresholds to include CpGs: *P* < 1 × 10^–1^, *P* < 1 × 10^–3^, *P* < 1 × 10^–5^. To estimate the variance explained by MS above genetic variants, polygenic scores (PGS) were created based on the summary statistics from the handedness GWAS of Cuellar-Partida et al.^13^ with exclusion of NTR, ALSPAC and 23andMe cohorts (*N*_*GWAS*_ = 196,419). Since four scores were tested (3 methylation scores, one polygenic score), we applied Bonferroni correction for four tests (*α* = 0.05/4 = 0.0125) (Supplementary Table 30, Supplementary Fig. 14). The results are summarized in Supplementary Table 31. MS did not predict left-handedness in NTR and ALSPAC adults, or in children at 7–9 years old and did not explain variance over and above the variance explained by the PGS in the combined model (R^2^_MS_ from -0.17 to 1.28%, R^2^_PGS_ from 0.002 to 0.46%). The largest amount of explained variance was in ALSPAC at 7 years old for the MS based on CpGs at *P* < 1 × 10^–5^ (R^2^_MS_ = 1.28%, *P* = 0.1, N_CpGs_ = 7).

## Discussion

We have performed an epigenome-wide association study of left-handedness, including left- and right-handed individuals from two population-based cohorts from the Netherlands and the UK. In the meta-analysis, combining the NTR and ALSPAC adult cohorts, two DMRs associated with left-handedness. The first DMR (genomic location: chr20q11.23, 36,148,679:36,149,022) is located within the 5’UTR of the BLCAP apoptosis inducing factor (*BLCAP*) gene and nearby the transcription start site (TSS1500) of the neuronatin (*NNAT*) gene. *BLCAP* encodes a protein that reduces cell growth by stimulating apoptosis. *NNAT* is located within intron of *BLCAP* and is involved in brain development and nervous system structure maturation and maintenance. *BLCAP* and *NNAT* are imprinted in the brain^49^. The second intron of *NNAT* regulates the expression of *BLCAP* transcripts acting as an imprint control region regulating also allele-specific DNA methylation and histone modifications^50^. Around 50% of CpGs in a region covering the *NNAT* CpG islands are methylated in brain and other tissues, suggestive of differential allele-specific methylation^51^. The imprinted DMR for these genes [chr20:36,139,941-36,159,190^52^] overlaps with the left-handedness DMR that we identified (chr20:36,148,679-36,149,022). A potential connection of genomic imprinting with handedness was previously suggested in a study of another imprinted gene *LRRTM1*^41^. CpGs from the handedness-associated region at chromosome 20 previously associated with myalgic encephalomyelitis and chronic fatigue syndrome, preterm birth, obesity, metabolic parameters, and arm fat mass (DXA scan measurement).

The second DMR (genomic location: chr2p25.1, 9,614,471:9,614,744) is located within the isoamyl acetate hydrolyzing esterase 1 (*IAH1*) gene. The *IAH1* gene encodes an acyl esterase and is associated with neonatal inflammatory skin and bowel disease, and a disease with an inborn error of leucine metabolism (3 methylglutaconic aciduria type 1). CpGs from the region previously associated with gestational age, bone mineral density, metabolic parameters, and schizophrenia. Some of these traits have been reported to be associated with handedness in epidemiological studies, e.g. BMI^53^ and gestational age^54^, for which we adjusted in our analyses. Previous analysis of the genetic correlations between left-handedness and 1349 complex traits using LD-score regression did not reveal any genetic correlations at FDR < 5%, but suggestive positive correlations were observed with neurological and psychiatric traits, including schizophrenia^13^.

Even though no DMPs were identified after correction for multiple testing, and effect sizes of top CpGs were small (mean differences between left- and right-handed individuals smaller than 1%), the high-ranking CpGs are of potential interest. The second-ranking CpG cg09239756 (genomic location: chr12, 106,642,360) is located near the cytoskeleton associated protein 4 (*CKAP4*) gene. This gene mediates the anchoring of the endoplasmic reticulum to microtubules. Microtubules are an important cytoskeleton component that play a role in neuronal morphogenesis and migration, and axon transport^31^. Microtubules have been widely discussed in association with handedness^15,29^ and brain anatomical asymmetry^32^, and genes involved in microtubule pathways were enriched in the GWAS of handedness^13^. Moreover, in our enrichment analysis, we found that CpGs located within a 1 Mb window from SNPs associated with left-handedness in the GWAS meta-analysis by Cuellar-Partida et al.^13^ were more strongly associated with left-handedness in our meta-analysis compared to CpGs outside of this window. Larger EWAS meta-analysis or replication in additional independent cohorts is necessary to establish the robustness of the top DMPs.

Hand movements together with other lateralized movements and molecular signs of lateralization are observed at very early stages of human development in the uterus^6–8^. Therefore, DNA methylation differences associated with hand preference are expected to emerge early in development. While DNA methylation at some CpGs in the genome changes throughout the lifespan^45^, the DNA methylation signal associated with left-handedness was moderately consistent from birth throughout the lifespan: DMP effects correlated in ALSPAC offspring from birth to 24 years old, although genome-wide significance for DMPs was not reached. Consistency in DNA methylation signal associated with left-handedness at different time-points may indicate that the pattern for left-handedness is conserved through the lifespan.

Several DMRs were detected in buccal cells in children around 9 years old (genomic locations: chromosomes 8, 22, 9, and 12) after correction for multiple testing. Annotation of these regions implicate the following protein coding genes: the plectin gene (*PLEC1)*, the myoglobin gene (*MB*) gene, the elongator complex protein 1 gene (*ELP1*), the actin binding transcription modulator gene (*ABITRAM*), and the WNK lysine deficient protein kinase 1 gene (*WNK1*). The CpGs in these regions are mostly hypomethylated in left-handed individuals. The genes encode for proteins that participate in cytoskeleton functions, chromatin organization, development of neurons, and metabolism. CpGs from DMRs in buccal cells previously associated with other phenotypes: CpGs from the DMR on chromosomes 8 with myalgic encephalomyelitis, chronic fatigue syndrome, multiple sclerosis, and gestational age; CpGs from the DMR on chromosome 9 with bone mineral density, tissue mass of the arm (DXA scans measurement), and multiple metabolic parameters. Interestingly, some of these phenotypes also associated with CpGs from our meta-analysis in blood DNA methylation.

A difference in handedness preference in MZ twin pairs has always fascinated parents of twins and twins themselves: how can children with almost identical genes differ for such a prominent trait? Handedness discordance in identical twins was described a long time ago^17,18^, and the percentage of MZ discordant twins were reported as 20% of 3486 MZ twins in East Flanders^19^, and 19% of 1724 MZ twins from a London twin study^28^. We observed that 21% of adult MZ twins and 24% of young MZ twins were discordant for handedness in our study, but we did not detect DNA methylation differences among them in blood or buccal cells. This null finding could mean that handedness discordance is not associated with methylation differences in the tissues that we studied (but might be present in other tissues), or that our analysis was underpowered to detect methylation differences associated with handedness discordance. Our discordant MZ twin analysis may be underpowered to detect small DNA methylation differences^55^, as it included only 133 MZ discordant adult twin pairs and 86 child twin pairs. Different, not mutually exclusive, hypotheses have been proposed for handedness discordance of MZ twins, including large unique environmental effects, that are deduced based on the low heritability of around 25% as estimated in the meta-analysis by Medland et al.^10^. McManus^16^ emphasizes that such unique environmental factors may represent what in biology is referred to as noise, randomness or fluctuating asymmetry. McManus quotes Mitchell^56^ saying that such processes are “caused not by any factors outside the organism, but by inherent variation in the processes of development”. These reflect developmental variance unique to the person. In a discussion and review of the human and animal studies literature Molenaar et al.^57^ called these processes ‘the third source of individual differences’, i.e. a third source besides genetic and environmental influences on individual differences and discuss how deterministic growth process may give rise to highly variable results.

There is a growing interest to improve the prediction of traits with use of other omics data than SNPs, like DNA methylation^37^ and by non-genetic early life factors (e.g. earlier factors associated with left-handedness including birth weight, being multiple, month of birth, breastfeeding etc.^58^). Given the low heritability of handedness (~ 25%^10,11^), it is expected that non-genetic factors play a role. Although together early life factors had minimal predictive value^58^, they may inspire the search for DNA methylation signatures, as DNA methylation signatures later in life were found for birthweight^47^. Single CpGs did not individually reach statistical significance in our EWAS, but combining information across multiple CpGs into an overall methylation score can be a more powerful approach to capture variation in handedness. We calculated methylation scores as weighted sums of the individual’s methylation loci beta values of a pre-selected number of CpG sites. However, the predictive value of polygenic and methylation scores for handedness was low, which likely reflects that current GWAS and EWAS analyses for handedness are still underpowered.

**Figure 3:**
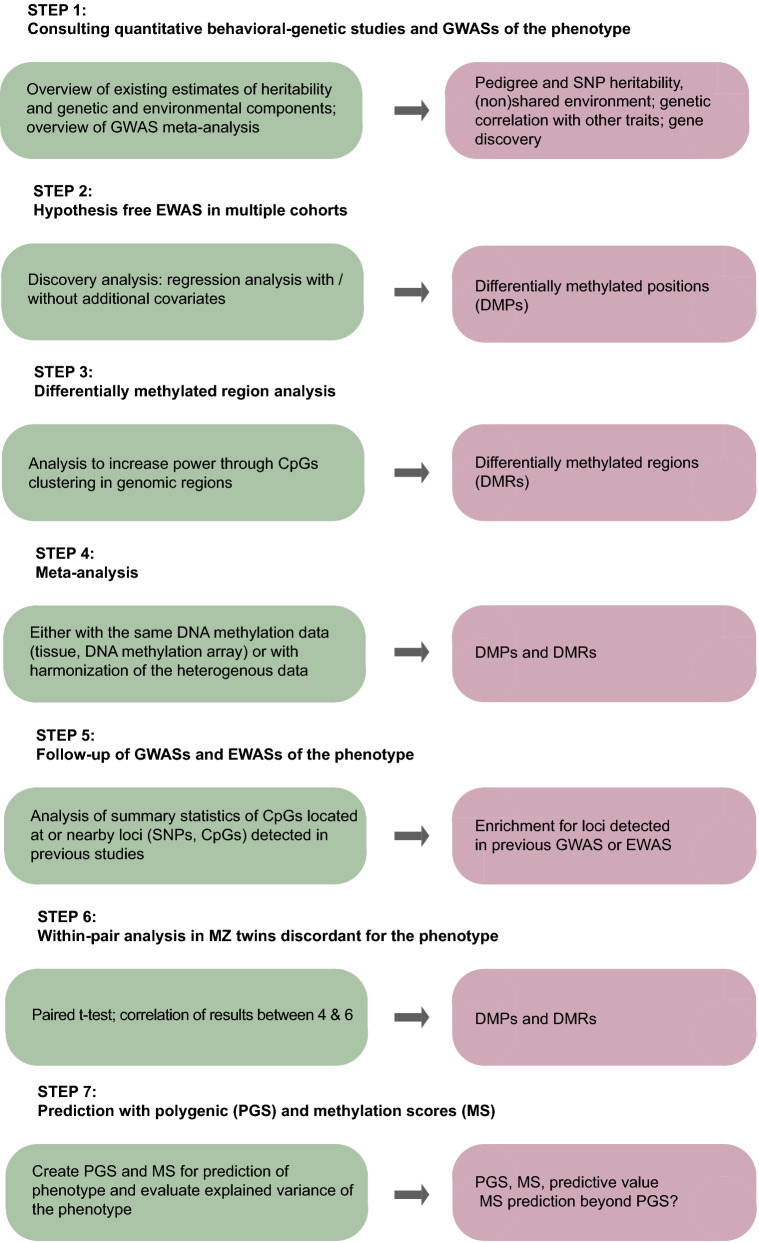
Seven-step approach to DNA methylation signature discovery, incorporating twin design. The figure represents the methodology of DNA methylation signature discovery study of a phenotype in multiple steps. It integrates behavioral-genetic and SNP-based methods (step 1) to estimate heritability, epigenome-wide study methods (steps 3–4) for association analyses, follow-up of results using summary statistics from previous EWASs and GWASs (step 5), the discordant twin design (step 6), and methods integrating polygenic and DNA methylation data (step 7) in enrichment analysis. Specific methods for each step are presented on the left and outcomes on the right. GWAS, genome-wide association study. EWAS, epigenome-wide association study. SNP, single nucleotide polymorphisms. CpG, cytosine-phosphate-guanine. PGS, polygenic scores. MS, methylation scores. DMPs, differentially methylated positions. DMRs, differentially methylated regions.

Our multi-cohort epigenome-wide association study can be summarized in several key steps presented in Fig. 3. We examined DNA methylation data in different tissues (whole blood, cord blood, buccal cells) and ages (from birth to adulthood). The limitations of the study are related to handedness measurements, available tissues, differences in platforms used for DNA methylation (Illumina 450 k, EPIC), and study power. There were slight differences in the assessment of left-handedness between NTR and ALSPAC. Power might increase by investigating a refinement of the handedness phenotype (e.g. hand skill measurement rather than self-report of the preferred hand), analysis of DNA methylation in more relevant tissues, and an increase in sample size. The difference in left-handedness rates among children born before and after 1960 may be due to a move away from being forced to use the right hand prior to 1960^59^. We accounted for this trend by including age (which correlates almost perfectly with birth year in these samples) as a covariate in the analyses, however, it should be noted that the forced use of the right hand in older generations may render the phenotype definition of handedness less precise. Our meta-analysis was based on whole blood methylation data, where the methylation level represents the overall level of DNA obtained from millions of white blood cells. We observed methylation differences between left- and right-handed individuals of up to 0.8% at top-DMPs. This small effect size could reflect that a methylation difference is present in only a sub-set of cells in an individual, or a sub-set of individuals in the population, or a combination. The biological implications of these findings remain to be established and our top-DMPs remain to be replicated in additional cohorts or larger meta-analysis. The primary tissues of interest for handedness are brain^2,29^, spinal cord^40^, and arm muscle tissues^4^, and the timing when these tissues are collected could also play a role, but the collections of these tissues are not widely available in population cohorts for obvious reasons. Although 4000 is considered a decent sample size for EWAS and smaller sample sizes have allowed for the successful detection of many loci where DNA methylation robustly associates with traits such as body mass index^38^, the effect sizes for handedness were unknown a priori and 4000 may still be too small to detect methylation differences associated with left-handedness. Similarly, sample sizes of EWASs of behavioral and psychiatric traits are now increasing beyond 10,000^60^. For GWAS of handedness that applied similar phenotyping as the current study, the increase of the sample size from about 400,000 to 1.7 million increased the number of associated loci from a handful to over forty^13^, and similar increases in the number of detected loci may occur when EWAS sample sizes for left-handedness increase. Finally, our study focused on DNA methylation, but other epigenetic processes could play a role in handedness such as histone modifications, post-translational regulation by miRNAs and X-chromosome inactivation that remain to be explored.

We reported an EWAS of left-handedness in two large population-based cohorts with data from children and adults, and examined performance of methylation scores and polygenic scores. Despite the plausible rationale of multiple genetic and non-genetic factors that may act via epigenetic pathways to influence the development of handedness, we did not uncover support for the hypothesis that DNA methylation in peripheral tissues captures much if any of the variation in handedness. We propose that future studies consider other tissues, such as related to central nervous system.

## Methods

### Overview

The primary epigenome-wide association study (EWAS) of left-handedness was performed in two cohorts with DNA methylation data in whole blood (Illumina, 450 k): NTR adults^61^ (*N* = 2682 individuals including twins, mean age at methylation 36.5, SD 12.7), and ALSPAC adults^62,63^ (*N* = 1232, mean age at methylation 48.98, SD 5.55). EWAS analyses were performed in each dataset separately, and summary statistics were combined in the meta-analysis (*N* = 3914) testing 409,563 CpGs. As this is a meta-analysis of existing DNA methylation datasets, no analyses were done to pre-determine sample sizes, but the sample size is larger compared to previously published DNA methylation studies of handedness^41,42^. We tested whether the EWAS signal was enriched in nearby loci detected in the previous GWAS on handedness^13^. Secondary analyses were performed in different tissues: in cord blood and peripheral blood in ALSPAC offspring^64^, i.e. the children of ALSPAC participants that contributed to the primary EWAS (N = 791 with DNA methylation data at birth, at 7, and 17 years old (Illumina 450 k chip), and/or at 24 years old (Illumina EPIC array)), and in buccal cells from an independent group of children from the NTR^65,66^ (N = 946 twins, mean age 9.5, SD 1.85, Illumina EPIC array). The number with DNA methylation and covariate data in ALSPAC differed at different time points from 442 to 759. We carried out within-pair twin analysis in NTR MZ twins discordant for handedness (*N*_*adults*_ = 133 twin pairs, *N*_*children*_ = 86 twin pairs). We performed EWAS analyses in each dataset and examined correlations between the regression coefficients of top CpGs (ranked on ascending *p*-value) from each EWAS analysis. Finally, we created and tested polygenic and DNA methylation scores for left-handedness. The study method and design are presented in Figs. 1 and 3. Detailed cohort information is provided in Appendix 1.

### Handedness

#### NTR

Information on hand preference for adults and children was collected by surveys and in small subgroups from laboratory-based projects. Parental reports on children were collected at 5 years and included seven items for different activities, from which the item “What hand does child use for drawing?” was selected. The four answer categories were left-handed, right-handed, both hands and do not know. Multiple adult surveys included the question: “Are you right-handed or left-handed?” (4 surveys) or “Are you predominantly left-handed or right-handed?” (3 surveys). The three answer categories were left-handed, right-handed, and both. For a small number of adult self-reports at younger ages (14, 16 or 18 years) or parental assessment at age 5 were also available.

#### ALSPAC

Adults (mothers and fathers) were asked which hand they used to write, draw, throw, hold a racket or bat, brush their teeth, cut with a knife, hammer a nail, strike a match, rub out a mark, deal from a pack of cards or thread a needle (11 questions). Child handedness was assessed at 42 months by questionnaire in which the mother was asked which hand the child used to draw, throw a ball, color, hold a toothbrush, cut with a knife, and hit things (6 questions). Responses were scored − 1, 0 or 1 for left, either or right, respectively. Handedness was coded as 1 for left-handed or 0 for right-handed in both cohorts.

### DNA methylation and genotyping

DNA methylation was measured with the Infinium HumanMethylation450 BeadChip Kit which measures more than 450,000 methylation sites (primary analysis in adults in NTR and ALSPAC and secondary analysis in ALSPAC offspring at birth, 7 and 17 years old), or the Infinium MethylationEPIC BeadChip which measures more than 850,000 methylation sites (secondary analysis in ALSPAC offspring at 24 years old and NTR children). Genotyping for polygenic risk scores was done on multiple platforms with imputation of the target data using reference haplotypes from 1000 genomes reference panel. Cohort-specific details on biosample collection, DNA methylation profiling, quality control, cell-type proportions measurements and genotyping are described in Appendix 1.

### Intergroup differences

We tested if there were differences in characteristics that were included in EWAS models (such as age at biological sample collection, sex, body mass index (BMI), smoking status at blood collection for adults, and gestational age, maternal smoking during pregnancy, birth weight for children, cell proportions/percentages in buccal swabs and in blood samples) between left- and right-handed individuals by generalized estimating equations (GEE) to accommodate the relatedness among the twins in NTR, and by standard logistic regression in ALSPAC. The R package ‘gee’ was used with the following specifications: binomial (for ordinal data) link function, 100 iterations, and the ‘exchangeable’ option to account for the correlation structure within families and within persons. Right- and lefthanded MZ discordant twins were compared with paired t-test for the traits that were not identical in twins (birth weight, BMI, smoking, cell percentages). All statistical tests here and below were two-tailed.

### Epigenome-wide association analyses

#### Primary analyses

The association between DNA methylation levels and left-handedness was tested for each site under a linear model (ALSPAC) or generalized estimating equation (GEE) model accounting for relatedness of twins (NTR). DNA methylation beta-values were the dependent variable and were typically normally distributed. The following predictors were included in the basic model: handedness (coded as 0 = right-handed and 1 = left-handed), sex, age at blood sampling, percentage of blood cells for blood samples, and technical covariates in NTR and ALSPAC (see Appendix 1). An adjusted model was fitted to account for BMI and smoking status at blood draw in both NTR and ALSPAC adult cohorts, because BMI and smoking have large effects on DNA methylation in adults^38,46^. The primary results reported in the paper are based on the fully adjusted model. The models are described in Appendix 2. Throughout the text, we refer to regression coefficients from the EWAS, which represent the methylation difference between left-handed and right-handed individuals on the methylation beta-value scale. A positive regression coefficient (*β*) means a higher methylation level in left-handed individuals. The value of an individual on the methylation scale is commonly also symbolized as beta (*β*) and ranges from 0 to 1, where 0 represents a methylation level of 0% and 1 represents a methylation level of 100%.

#### Secondary analyses

The same basic models were fitted to the data from ALSPAC and NTR children. For DNA methylation in buccal cells, cell proportions (epithelial cells, natural killer cells) for buccal samples were included instead of percentage of blood cells. As several characteristics, such as gestational age and birthweight, affect DNA methylation^48,67^, we included these in the adjusted model in children (see Appendix 2).

In the within-pair analysis of discordant MZ twins, paired t-tests were employed to test for methylation differences between the left-handed and the right-handed twins. Paired t-tests were performed in R on residual methylation levels, which were obtained by adjusting the DNA methylation *β*-values for sample plate, array row, cell proportions in buccal samples in children and sample plate, array row, and percentages of blood cells in adults. Additional covariates, birth weight in children and BMI and smoking status in adults, were added in adjusted model. Age, sex, maternal smoking, and gestational age were not included because these variables are identical in MZ twins.

To account for multiple testing, we considered Bonferroni correction and a False Discovery Rate (FDR) of 5%. The Bonferroni corrected p-value threshold was calculated by dividing 0.05 by the number of genome-wide CpGs tested, and false discovery rate (FDR) q-values were computed with the R package ‘qvalue’ with default settings. The Bayesian inflation factor (*λ*) was calculated with the R package Bacon^68^ (see Supplementary Table 5).

### Meta-analysis

A meta-analysis was performed in METAL^69^ based on estimates (regression coefficients) and standard errors from the EWAS of handedness performed with GEE in NTR and linear regression in ALSPAC. NTR and ALSPAC adult cohorts were combined. In total, 409,563 CpG sites present in both cohorts were tested with statistical significance evaluated after Bonferroni correction and at an FDR *q*-value < 0.05.

### Comparison of top CpGs from different analyses

To compare top CpGs from different analyses, we repeated the NTR EWAS analyses in adults in children and meta-analysis with discordant MZ twin pairs removed to avoid sample overlap. We selected methylation sites that overlapped in 13 analyses with adjusted model (meta-analysis, meta-analysis without discordant MZ twins, EWAS NTR adults, EWAS NTR adults without discordant MZ twins, EWAS ALSPAC adults, EWASs ALSPAC at birth, 7, 17, 24 years, EWAS NTR children, EWAS NTR children without discordant twins, and within-pair analyses of discordant MZ twin adults and children) that resulted in 379,924 methylation sites. We calculated Pearson correlations for effect estimates of the top 100 CpGs ranked by *p*-value from one analysis with the effect estimates of the same CpGs in other analyses. Statistical significance of correlations was assessed after Bonferroni correction for the number of correlations tested: α = 0.05/(13 × 13 − 13) = 0.0003.

### Differentially methylated regions

We used the R dmrff library^36^ for R to identify regions where CpG sites showed evidence for association with handedness. Dmrff identifies DMRs by meta-analysing EWAS summary statistics from CpG sites in each region while adjusting for dependencies between the sites and uncertainty in the EWAS effects (https://github.com/perishky/dmrff). In this study, dmrff was applied separately in the NTR and ALSPAC cohorts, and then used to identify DMRs in common between the cohorts by meta-analysis. As previously described^36^, DMR meta-analysis preceded by first identifying candidate DMRs using the EWAS meta-analysis summary statistics, calculating DMR statistics for these candidates in each cohort separately, and then meta-analysing the DMR statistics across the two cohorts. The DMR effect size is a weighted sum of the EWAS effects for each CpG site (i.e. methylation differences between LH and RH). All dmrff p-values were adjusted for multiple tests (Bonferroni adjustment) by multiplying them by the total number of DMRs considered. We report significant regions (*P*_*adj*_ < 0.05) including at least two CpG sites within a 500 bp window observed to be nominally associated with handedness by EWAS (*P* < 0.05). The average absolute DNA methylation difference in the region between left-handers and right-handers is calculated as the sum of absolute regression coefficients of each CpG in the region divided by the number of CpGs. We plotted the DMRs with the coMET R Bioconductor package^70^ to graphically display additional information on physical location of CpGs, correlation between sites, statistical significance, and functional annotation (annotation tracks included genes Ensembl, CpG islands (UCSC), regulation Ensembl).

### GWAS follow-up

GWAS follow-up analyses were performed to examine whether CpGs within a 1 Mb window of loci detected by the GWAS for left-handedness^13^, on average, showed a stronger association with left-handedness than other genome-wide methylation sites (Infinium HumanMethylation450 BeadChip). We obtained a SNP list based on the GWAS meta-analysis without NTR, ALSPAC, and 23andMe by Cuellar-Partida et al.^13^ (196,419 individuals, N_SNPs_ = 13,550,404), from which we selected all SNPs with a *P*-value < 1.0 × 10^–08^, < 1.0 × 10^–06^, and < 1.0 × 10^–05^, and determined the distance of each Illumina 450 k methylation site to each SNP. To test whether methylation sites near GWAS loci were more strongly associated with left-handedness, meta-analysis EWAS test statistics were regressed on a variable indicating if the CpG is located within a 1 Mb window from SNPs associated with handedness (1 = yes, 0 = no):

$$\left| {Z{\text{score}}} \right| = {\text{Intercept}} + \beta_{{\text{category x}}} \times {\text{Category}}\,{\text{x}},$$

where |Zscore| represents the absolute Zscore for a CpG from the EWAS meta-analysis of handedness; β_category x_ represents the estimate for category *x*, i.e. the change in the EWAS test statistic associated with a one-unit change in category *x* (e.g. being within 1 Mb of SNPs associated with left-handedness). For each enrichment test, bootstrap standard errors were computed with 2000 bootstraps with the R-package “simpleboot”. Statistical significance was assessed at *α* = 0.05. As control analysis, the same follow-up was performed using GWAS summary statistics on a trait that is unrelated to handedness type 2 diabetes in UK Biobank cohort (N = 244,890) ^43^. GWAS summary statistics were downloaded from GWASAtlas (https://atlas.ctglab.nl/traitDB/3686; 41204_E11_logistic.EUR.sumstats.MACfilt.txt; accessed on February 1 2021).

We looked up CpG sites associated with handedness-associated SNPs with a *P*-value < 1.0 × 10^–08^ (based on the GWAS meta-analysis by Cuellar-Partida et al.^13^ without 23andMe, NTR and ALSPAC, resulting in a list of 420 SNPs) using the mQTL database maintained by the Genetics of DNA Methylation Consortium (GoDMC, N = 27,750 European samples; http://mqtldb.godmc.org.uk/about)^44^. We then checked if associations with handedness were observed for these sites in our EWAS meta-analysis and DMR meta-analysis.

#### EWAS follow-up

To examine previously reported associations for epigenome-wide significant DMRs associated with left-handedness in our study, we looked up CpGs from the regions in the EWAS Atlas^71^ (https://bigd.big.ac.cn/ewas/tools; accessed on August 1 2020) and EWAS catalogue^72^ (http://www.ewascatalog.org; access on November 1 2020).

### Polygenic and methylation scores

*Polygenic scores (PGS)* for handedness were calculated based on the GWAS meta-analysis without 23andMe by Cuellar-Partida et al.^13^. To avoid overlap between the discovery and target samples, summary statistics without NTR and ALSPAC were requested (196,419 individuals, N_SNPs_ = 13,550,404). The linkage disequilibrium (LD) weighted betas were calculated using a LD pruning window of 250 KB, with the fraction of causal SNPs set at 0.50 by LDpred^73^. We randomly selected 2500 2nd degree unrelated individuals from each cohort as a reference population to calculate the LD patterns. The resulting betas were used to calculate the PGSs in each dataset using the PLINK 1.9 software. All PGSs were standardized (mean of 0 and standard deviation of 1). *Methylation scores (MS)* were calculated in NTR based on EWAS summary statistics obtained from ALSPAC, and vice versa, as previously done to create methylation scores for BMI and height^74^. We calculated same-tissue same-age DNA-methylation scores based on methylation data from NTR adults (blood) and ALSPAC parents (blood), and cross-tissue DNA-methylation scores based on data from NTR and ALSPAC offspring, with DNA methylation measured in buccal cells, and blood, respectively (see Fig. 1). For each individual, a weighted score sum was calculated for left-handedness by multiplying the methylation value at a given CpG by the effect size of the CpG (β), and then summing these values over all CpGs: DNA methylation score (i) = β_1_*CpG_1i_ + β_2_*CpG_2i_··· + β_n_*CpG_ni_, where CpG_n_ is the methylation level at CpG site *n* in participant *i*, and *β*_*n*_ is the regression coefficient at CpG_n_ taken from summary statistics of the EWAS analysis. All methylation scores were standardized (mean of 0 and standard deviation of 1). We used weights from summary statistics of EWASs in four cohorts: NTR adults, ALSPAC adults, NTR children, ALSPAC offspring at 7 years old. Subsets of CpGs to be included in methylation scores were selected based on *P*-value < 1 × 10^–1^, < 1 × 10^–3^, and < 1 × 10^–5^. We analysed the predictive value of the left-handedness polygenic scores and methylation scores in NTR and ALSPAC adult and child cohorts from our EWAS study. To quantify the variance explained by the PGS and MS, we used the approach proposed by Lee et al.^75^, where coefficients of determination (R^2^) for binary responses are calculated on the liability scale. The equations of all models are provided in Appendix 2. Statistical significance was assessed following Bonferroni correction for the number of scores tested (PGS and 3 MSs). This resulted in *α* = 0.05/4 = 0.0125, nominal significance at 0.05.

#### Ethics statement

All methods were performed in accordance with the Declaration of Helsinki. For *NTR,* the study was approved by the Central Ethics Committee on Research Involving Human Subjects of the VU University Medical Centre, Amsterdam, an Institutional Review Board certified by the U.S. Office of Human Research Protections (IRB Number IRB00002991 under Federal-wide Assurance FWA00017598; IRB/institute codes, NTR 03-180). All subjects provided written informed consent. For children, written informed consent was given by their parents. For *ALSPAC,* ethical approval for the study was obtained from the ALSPAC Ethics and Law Committee and the Local Research Ethics Committees. All subjects provided written informed consent. For children, written informed consent was given by their mothers.

## Supplementary Information

Supplementary Information 1.

Supplementary Information 2.

Supplementary Information 3.

Supplementary Information 4.

Supplementary Information 5.

Supplementary Information 6.

## Data Availability

The HumanMethylation450 BeadChip data from the NTR are available as part of the Biobank-based Integrative Omics Studies (BIOS) Consortium in the European Genome-phenome Archive (EGA), under the accession code EGAD00010000887 (https://ega-archive.org/datasets/EGAD00010000887). The Infinium MethylationEPIC from NTR are available from the Netherlands Twin Register on reasonable request (https://tweelingenregister.vu.nl/information_for_researchers/working-with-ntr-data). DNA methylation data from ALSPAC are available at ALSPAC and can be provided on request. The study website contains details of all the data that is available through a fully searchable data dictionary and variable search tool (http://www.bristol.ac.uk/alspac/researchers/our-data). The code used to perform the primary and secondary analyses is available at https://github.com/MRCIEU/handedness-ewas. The pipeline for the DNA methylation array analysis developed by the Biobank-based Integrative Omics Study (BIOS) consortium are available here: https://molepi.github.io/DNAmArray_workflow/. EWAS summary statistics for the top 100 CpGs are given in Supplemental Tables 6–11 and 15–29. The full EWAS summary statistics from the meta-analysis with basic and adjusted model are provided in Supplemental Tables 32 and 33. The full summary statistics for all other analyses are available upon request from the corresponding author.
