# Supplementary material for: DNA methylation in peripheral tissues and left-handedness
Source: Sci Rep. 2022 Apr 4;12:5606. doi: 10.1038/s41598-022-08998-0 (PMC8980054; doi:10.1038/s41598-022-08998-0)
Supplement: Supplementary file 2 — Supplementary Information 2. [file 41598_2022_8998_MOESM2_ESM.pdf]

Scientific Reports

SUPPLEMENTARY INFROMATION

DNA methylation in peripheral tissues and Left-handedness

Veronika V. Odintsova, Matthew Suderman, Fiona A. Hagenbeek, Doretta Caramaschi, Jouke-Jan Hottenga, René Pool, BIOS consortium, Conor V. Dolan, Lannie Ligthart, Catharina E.M. van Beijsterveld, Gonneke Willemsen, Eco J.C. de Geus, Jeffrey J. Beck, Erik A. Ehli, Gabriel Cuellar-Partida, David M. Evans, Sarah E. Medland, Caroline L. Relton, Dorret I. Boomsma, Jenny van Dongen

Content

Appendix 1. Cohort specific methods 2

Appendix 2. EWAS Model Equations 8

Supplementary Table 1. Primary analysis group characteristics 11

Supplementary Table 2. Year of birth and handedness in NTR adults 11

Supplementary Table 3. Secondary analysis group characteristics 12

Supplementary Table 4. Characteristics of discordant MZ twins 13

PART 1. PRIMARY ANALYSIS 14

Supplementary Figure 1. QQ plots of EWAS results on left-handedness in primary analysis 14

Supplementary Figure 2. DNA methylation level at top CpGs from meta-analysis in right- and left-handed in NTR 15

Supplementary Figure 3. DNA methylation level at top CpGs from meta-analysis in right- and left-handed in ALSPAC ..16

Supplementary Figure 4. DNA methylation level at CpGs from left-handedness associated DMR at chromosome 20 in right- and left-handed in NTR 17

Supplementary Figure 5. DNA methylation level at CpGs from left-handedness associated DMR at chromosome 20 in right- and left-handed in ALSPAC 18

Supplementary Figure 6. DNA methylation level at CpGs from left-handedness associated DMR at chromosome 2 in right- and left-handed in NTR 19

Supplementary Figure 7. DNA methylation level at CpGs from left-handedness associated DMR at chromosome 2 in right- and left-handed in ALSPAC 20

Supplementary Table 14. GWAS follow-up results 21

Supplementary Figure 8. QQ plots of p-values of CpGs located within 1Mb window from GWAS SNPs associated with left-handedness and type 2 diabetes 21

PART 2. SECONDARY ANALYSIS 22

Supplementary Figure 9. QQ plots of EWAS results on left-handedness in secondary analysis 22

Supplementary Figure 10. Correlations among the effects of top 100 CpGs across analyses 23

Supplementary Figure 11. Overlaps in top CpGs across analyses 24

Supplementary Figure 12. Scatterplot of effects in ALPAC adults and ALSPAC at birth 24

Supplementary Figure 13. Differentially methylated regions associated with left-handedness in secondary analysis 25

Supplementary Table 30. Polygenic and methylation scores general description 26

Supplementary Figure 14. Polygenic and methylation scores histograms 26

Supplementary Table 31. Performance of left-handedness polygenic and methylation scores 27

Supplementary Table 32. Meta-analysis summary statistics (basic model) (legend) 28

Supplementary Table 33. Meta-analysis summary statistics (adjusted model) (legend) 28

Appendix 1. Cohort specific methods

Netherlands Twin Register (NTR)

Subjects and samples

Primary analysis. The adult NTR Biobank cohort[1] included twins, parents of twins, siblings of twins and spouses of twins, and had DNA methylation data from blood samples, as previously described[2]. Complete data on handedness and DNA methylation from blood samples were available for 2,682 individuals with mean age 36.5 years (SD=12.7, age range 17-79), of whom 2,486 were twins/triplets and 196 were parents, siblings or spouses of twins. All subjects provided written informed consent. The study was approved by the Central Ethics Committee on Research Involving Human Subjects of the VU University Medical Centre, Amsterdam, an Institutional Review Board certified by the U.S. Office of Human Research Protections (IRB number IRB00002991 under Federal-wide Assurance FWA00017598; IRB/institute codes, NTR 03-180).

Secondary analysis. The NTR child cohort included in the EWAS of buccal cell DNA was part of a project on childhood aggression "Aggression in Children: Unraveling gene-environment interplay to inform Treatment and InterventiON strategies" (ACTION)[3,4]. The ACTION-NTR cohort[5] included 1,235 children for whom epigenome-wide data was successfully assessed, mainly from MZ twin pairs. ACTION included twins who at least once scored high or low on a test score for aggression from the population-based NTR. Complete data on left-handedness and DNA methylation from buccal samples were available for 946 twin individuals (mean age=9.5, SD=1.8, range=5-12). The study was approved by the Central Ethics Committee on Research Involving Human Subjects of the VU University Medical Centre, Amsterdam, an Institutional Review Board certified by the U.S. Office of Human Research Protections (IRB number IRB00002991 under Federal-wide Assurance FWA00017598; IRB/institute codes, NTR 03-180). For children, written informed consent was given by their parents. The ancestry was European (Dutch) in NTR groups.

Handedness measurement

Information on hand preference for adults and children was collected by surveys and in small subgroups from laboratory-based projects. Parental reports on children were collected at 5 years and included 7 items for different activities, from which the item "What hand does child use for drawing?" was selected. The four answer categories were left-handed, right-handed, both hands and do not know. Multiple adult surveys included the question: "Are you right-handed or left-handed? (4 surveys)" or "Are you predominantly left-handed or right-handed?" (3 surveys). The three answer categories were left-handed (LH), right-handed (RH), and both. For a small number of adults, information came from self-reports at younger ages (14-18 years) or parental assessment at age 5.

For children, there were 967 subjects with DNA methylation in buccal cells and handedness data. We included 807 right-handed and 139 left-handed children, and excluded children with 'both hands' and 'don't know' responses (10 and 1, respectively).

For adults, there were 2,836 participants with DNA methylation in blood and handedness data. We included 2,358 right-handed and 324 left-handed adults and excluded 75 mixed-handed subjects, and 13 / 64 left- / right-

handers with missing information on buccal cell counts. For the group of 2,682 included persons (see "Reliability information for handedness in NTR adults" below), there were 2,434 participants with information from one or multiple adult surveys; 74% had completed more than 1 survey and 25% more than 2 surveys. The consistency across surveys was 98% (1,750 out of 1,788), the remaining 38 participants had consistent replies after removal of one deviating answer. The 248 subjects without adult surveys were recruited into the NTR as children and information on their handedness comes from multiple surveys (except for 7 subjects), that were completed by their parents, and from self-reports between ages 14 and 18. For 18 persons one survey was available, all others had information on more than one survey. For 13 subjects the information was inconsistent across surveys, they were assigned the assessment based on the survey information collected at age 5 from the item on "drawing". For the 7 persons without survey data, information on handedness was collected at laboratory-based assessment when they were 16 years old and took part in an EEG study on brain function.

Reliability information for handedness in NTR adults

	N	Percent in total sample
**Total**	**2682**	**100**
**Handedness based on Adult NTR**	**2434**	**90.8**
Number of surveys		
one survey	646	24.1
from 2 to 5 surveys	1788	66.7
Reliability information for handedness		
one measurement	646	24.1
consistent across surveys	1750	65.2
consistent after removal of 1 survey	38	1.4
**Handedness based on Young NTR (5-18 years old)**	**248**	**9.2**
Number of surveys		
one survey	18	0.7
from 2 to 5 surveys	223	8.3
lab-based project	7	0.3
Reliability information for handedness		
one measurement (YNTR or lab-based)	25	0.9
consistent across all surveys/measurements	185	6.9
consistent after removal of 1 survey	25	0.9
inconsistent, information at YNTR at 5 years old is used	13	0.5

Methylation measurements

NTR adults. The NTR blood DNA methylation data was generated as part of the Biobank-based Integrative Omics Study (BIOS) consortium[2,6]. Blood collection procedures were described previously[1]. DNA methylation was assessed with the Infinium HumanMethylation450 BeadChip Kit (Illumina, San Diego, CA, USA), wet laboratory procedure, preprocessing analyses, and quality control were performed at the Human Genotyping facility (HugeF) of ErasmusMC, the Netherlands (http://www.glimdna.org/) and have been described previously[2,6]. Only the autosomal methylation sites were analyzed, i.e., 411,169 methylation sites. The percentages of neutrophils, monocytes and eosinophils were used to adjust DNA methylation data for inter-individual variation in white blood cell proportions[2]. Missing probe values (probes with missing values in over 5% of the sample had been removed) were imputed with the function imputePCA from the package missMDA as implemented in the pipeline for DNA methylation array analysis developed by the BIOS consortium[7].

NTR children. DNA samples were collected from buccal swabs, as previously described[1]. DNA methylation was measured using the Infinium MethylationEPIC BeadChip (Illumina, San Diego, CA, USA)[9], wet laboratory procedure, preprocessing analyses, and quality control were performed by the Human Genotyping facility (HugeF) of ErasmusMC, the Netherlands (http://www.glimdna.org/), as previously described[10]. Only autosomal methylation sites were analyzed, leaving 787,711 out of 865,859 sites for analysis. Cellular proportions of buccal cells were estimated from DNA methylation profiles using the deconvolution algorithm HepiDISH[11]. Cell proportions of epithelium cells and natural killer cells were included in statistical models to adjust for cellular heterogeneity. DNA methylation outliers were identified as those three times the inter-quartile range from the nearest of the first and third quartiles. Outliers were replaced with missing values.

Genotyping

Genotyping in NTR was done on multiple platforms including Perlegen-Affymetrix, Affymetrix 6.0, Affymetrix Axiom, Illumina Human Quad Bead 660, Illumina Omni 1M and Illumina GSA. Quality control was carried out and haplotypes were estimated using PLINK[12]. For each genotype platform, samples were removed if DNA sex did not match the expected phenotype, if the PLINK heterozygosity F statistic was < -0.10 or > 0.10, or if the genotyping call rate was < 0.90. SNPs were removed if the MAF $< 1\times10^{-6}$, if the Hardy-Weinberg equilibrium p-value was $< 1\times10^{-6}$, and/or if the call rate was < 0.95. Subsequently, for each platform, the genotype data was aligned with the 1000 Genomes reference panel using the HRC and 1000 Genomes checking tool, which tests and filters for SNPs with allele frequency differences larger than 0.20 as compared to the CEU population, palindromic SNPs and DNA strand issues. The data of the six platforms was then merged into a single dataset, keeping all quality-controlled SNPs of each platform. For each individual, one platform was chosen. Based on the ~10.8k SNPs that all platforms have in common, DNA Identity By Descent state was estimated for all individual pairs using the Plink and King programs. CEU population outliers, based on per platform 1000 Genomes PC projection with the Smartpca software[13], were removed from the data. Data were phased per platform using Eagle, and then imputed to 1000 Genomes and Topmed using Minimac following the Michigan imputation server protocols[14]. For the polygenic scoring the imputed data were converted to best guess data, and were filtered to include only ACGT SNPs, SNPs with MAF > 0.01, HWE p $> 10^{-5}$ and a genotype call rate > 0.98, and to exclude SNPs with more than 2 alleles. All Mendelian errors were set to missing. 20 PCs were calculated with Smartpca using LD-pruned 1000 Genomes–imputed SNPs that were also genotyped on at least one platform, had MAF > 0.05 and were not present in the long-range LD regions.

Avon Longitudinal Study of Parents and Children (ALSPAC)

Subjects and samples

The ALSPAC cohort[15–17] is a population-based birth cohort. All pregnant women living in the geographical area of Avon (UK) with expected delivery date between 1 April 1991 and 31 December 1992 were invited to participate. Approximately 85% of the eligible population enrolled, totaling 14,541 pregnant women who gave informed and written consent. The study website contains details of all the data that are available through a fully

searchable data dictionary and variable search tool (http://www.bris.ac.uk/alspac/researchers/data-access/data-dictionary/). The ALSPAC adult group comprised parents, including 1,232 mothers and fathers with mean age 48.98 years (SD=5.55, age range=31-75) when blood samples were acquired.

The ALSPAC child group (offspring of ALSPAC adult group) comprised 791 individuals recruited from birth who had information on handedness and DNA methylation profiles. DNA methylation from peripheral blood cells measured at different ages within the Accessible Resource for Integrated Epigenomics Studies (ARIES) project[18]: at birth (*N*=703), at mean age 7.44 (*N*=757), at mean age 17.11 (*N*=759), and at mean age 24.3 (*N*=442). Study data were collected and managed using REDCap electronic data capture tools hosted at the University of Bristol[19]. REDCap (Research Electronic Data Capture) is a secure, web-based software platform designed to support data capture for research studies. The ancestry was mainly European (UK). Ethical approval for the study was obtained from the ALSPAC Ethics and Law Committee and the Local Research Ethics Committees.

Handedness measurement

Adults (mothers and fathers) were asked which hand they used to write, draw, throw, hold a racket or bat, brush their teeth, cut with a knife, hammer a nail, strike a match, rub out a mark, deal from a pack of cards or thread a needle (11 questions). Responses were scored -1, 0 or 1 for left, either or right, respectively. Those with score sums from -11 to -7 were labeled left-handed (LH) and those with sums from 7 to 11 were labeled right-handed (RH). Individuals with scores outside these ranges were labeled ambidextrous or mixed-handed and excluded from this study.

Child handedness was assessed at 42 months by questionnaire in which the mother was asked which hand the child used to draw, throw a ball, color, hold a toothbrush, cut with a knife, and hit things (6 questions). Responses were scored -1, 0 or 1 for left, either or right, respectively. Those with score sums from -6 to -4 were labelled left-handed and those with sums from 4 to 6 were labelled right-handed.

Methylation measurements

The ALSPAC blood collection were generated at different ages. DNA methylation was measured with the Infinium HumanMethylation450 BeadChip Kit and Infinium MethylationEPIC BeadChip (Illumina, San Diego, CA, USA) as part of the ARIES[18]. Wet laboratory procedures, preprocessing analyses, and quality control were performed at the University of Bristol, as previously described[18]. Only autosomal probes were analysed in our study: 838,019 probes (Illumina EPIC human methylation arrays) at 24 years of age, and 471,465 probes (Illumina human methylation 450k arrays) at other ages. Blood cell-type proportions were estimated from DNA methylation profiles using deconvolution algorithms[20], and included in statistical models to adjust for cell type heterogeneity. Batch effects and additional unknown confounding were estimated using surrogate variable analysis (SVA) in *meffil*[21]. DNA methylation outliers were identified as those three times the inter-quartile range from the nearest of the first and third quartiles. Outliers were replaced with missing values.

Genotyping

Genetic data were collected from the blood samples obtained in clinic visits. Genotyping was conducted with the Illumina HumanHap550 quad chip for children and the Illumina human660W-quad array for mothers. Quality control measures were carried out and haplotypes estimated using ShapeIT. A phased version of the 1000 genomes reference panel from the Impute2 reference data repository was used, and imputation of the target data was performed with this, using all reference haplotypes. Following imputation, variants were retained only given info scores > 0.8 and minor allele frequency > 0.01. Retained variants were then converted to best-guess genotype calls. To avoid potential confounding due to relatedness, closely related individuals were removed using GCTA with a GRM cutoff of 0.05.

References

1. Willemsen, G. *et al.* The Netherlands Twin Register Biobank: A Resource for Genetic Epidemiological Studies. *Twin Res. Hum. Genet.* **13**, 231–245 (2010).
2. van Dongen, J. *et al.* Genetic and environmental influences interact with age and sex in shaping the human methylome. *Nat. Commun.* **7**, 11115 (2016).
3. Boomsma, D. I. *et al.* Aggression in children: unravelling the interplay of genes and environment through (epi)genetics and metabolomics. *J. Pediatr. Neonatal Individ. Med.* **4**, (2015).
4. Bartels, M. *et al.* Childhood aggression and the co-occurrence of behavioural and emotional problems: results across ages 3–16 years from multiple raters in six cohorts in the EU-ACTION project. *Eur. Child Adolesc. Psychiatry* **27**, 1105–1121 (2018).
5. Hagenbeek, F. A. *et al.* Urinary Amine and Organic Acid Metabolites Evaluated as Markers for Childhood Aggression: The ACTION Biomarker Study. *Front. Psychiatry* **11**, (2020).
6. Bonder, M. J. *et al.* Disease variants alter transcription factor levels and methylation of their binding sites. *Nat. Genet.* **49**, 131–138 (2017).
7. Sinke, L., van Iterson, M., Cats, D., Slieker, R. & Heijmans, B. DNAmArray: Streamlined workflow for the quality control, normalization, and analysis of Illumina methylation array data. (2019). doi:10.5281/ZENODO.3355292
8. Meulenbelt, I., Droog, S., Trommelen, G. J., Boomsma, D. I. & Slagboom, P. E. High-yield noninvasive human genomic DNA isolation method for genetic studies in geographically dispersed families and populations. *Am. J. Hum. Genet.* **57**, 1252–4 (1995).
9. Moran, S., Arribas, C. & Esteller, M. Validation of a DNA methylation microarray for 850,000 CpG sites of the human genome enriched in enhancer sequences. *Epigenomics* **8**, 389–399 (2016).
10. van Dongen, J. *et al.* DNA methylation signatures of aggression and closely related constructs: A meta-analysis of epigenome-wide studies across the lifespan. *Mol. Psychiatry* (2021). doi:10.1038/s41380-020-00987-x
11. Zheng, S. C. *et al.* A novel cell-type deconvolution algorithm reveals substantial contamination by immune cells in saliva, buccal and cervix. *Epigenomics* **10**, 925–940 (2018).
12. Purcell, S. *et al.* PLINK: a tool set for whole-genome association and population-based linkage analyses. *Am. J. Hum. Genet.* **81**, 559–575 (2007).
13. Patterson, N., Price, A. L. & Reich, D. Population structure and eigenanalysis. *PLoS Genet.* **2**, e190 (2006).
14. Das, S. *et al.* Next-generation genotype imputation service and methods. *Nat. Genet.* **48**, 1284–1287 (2016).
15. Boyd, A. *et al.* Cohort Profile: The 'Children of the 90s'—the index offspring of the Avon Longitudinal Study of Parents and Children. *Int. J. Epidemiol.* **42**, 111–127 (2013).
16. Fraser, A. *et al.* Cohort Profile: The Avon Longitudinal Study of Parents and Children: ALSPAC mothers cohort. *Int. J. Epidemiol.* **42**, 97–110 (2013).
17. Northstone, K. *et al.* The Avon Longitudinal Study of Parents and Children (ALSPAC): an update on the enrolled sample of index children in 2019. *Wellcome open Res.* **4**, 51 (2019).
18. Relton, C. L. *et al.* Data Resource Profile: Accessible Resource for Integrated Epigenomic Studies (ARIES). *Int. J. Epidemiol.* **44**, 1181–90 (2015).
19. Harris, P. A. *et al.* Research electronic data capture (REDCap)--a metadata-driven methodology and workflow

process for providing translational research informatics support. *J. Biomed. Inform.* **42**, 377–381 (2009).
20. Houseman, E. A. *et al.* DNA methylation arrays as surrogate measures of cell mixture distribution. *BMC Bioinformatics* **13**, 86 (2012).
21. Leek, J. T. & Storey, J. D. Capturing Heterogeneity in Gene Expression Studies by Surrogate Variable Analysis. *PLoS Genet.* **3**, e161 (2007).

Appendix 2. EWAS Model Equations

Primary and secondary analyses EWAS

The following models were fitted in each cohort in primary and secondary analyses. NTR: GEE. ALSPAC: linear regression.

<u>Basic model:</u>

NTR

For DNA methylation in peripheral blood in adults

$$CpGi = \alpha + \beta_{handedness} \text{ x left-handedness} + \beta_{sex} \text{ x sex} + \beta_{age} \text{ x age} + \beta_{Neu} \text{ x Neu} + \beta_{Eos} \text{ x Eos} + \beta_{Mono} \text{ x Mono} + \beta_{arrayrow} \text{ x array row} + \beta_{sampleplate2} \text{ x sample plate 2… + …} \beta_{sampleplate\ N} \text{ x sample plate N} + \varepsilon$$

For DNA methylation in buccal cells in children

$$CpGi = \alpha + \beta_{handedness} \text{ x handedness} + \beta_{sex} \text{ x sex} + \beta_{age} \text{ x age} + \beta_{Epi} \text{ x Epi} + \beta_{NK} \text{ x NK} + \beta_{arrayrow} \text{ x array row} + \beta_{sampleplate2} \text{ x sample plate 2… + …} \beta_{sampleplate\ N} \text{ x sample plate N} + \varepsilon$$

ALSPAC

For DNA methylation in peripheral blood in children and adults

$$CpGi = \alpha + \beta_{handedness} \text{ x left-handedness} + \beta_{sex} \text{ x sex} + \beta_{age} \text{ x age} + \beta_{B\ lym} \text{ x B lym} + \beta_{CD4T} \text{ x CD4T} + \beta_{CD8T} \text{x CD8T} + \beta_{NK} \text{ x NK} + \beta_{NK} \text{ x NK} + \beta_{Mono} \text{ x Mono} + \beta_{Gran} \text{ x Gran} + \beta_{surrogate\ variable2} \text{ x surrogate variable 2 +….} \beta_{surrogate\ variableN} \text{ x surrogate variable N} + \varepsilon$$

For DNA methylation in cord blood

$$CpGi = \alpha + \beta_{handedness} \text{ x left-handedness} + \beta_{sex} \text{ x sex} + \beta_{age} \text{ x age} + \beta_{B\ lym} \text{ x B lym} + \beta_{CD4T} \text{ x CD4T} + \beta_{CD8T} \text{x CD8T} + \beta_{NK} \text{ x NK} + \beta_{NK} \text{ x NK} + \beta_{Mono} \text{ x Mono} + \beta_{Gran} \text{ x Gran} + \beta_{nRBC} \text{ x nRBC} + \beta_{surrogate\ variable2} \text{ x surrogate variable 2 +….} \beta_{surrogate\ variableN} \text{ x surrogate variable N} + \varepsilon$$

<u>Adjusted model:</u>

NTR

For DNA methylation in peripheral blood in adults

$$CpGi = \alpha + \beta_{handedness} \text{ x left-handedness} + \beta_{sex} \text{ x sex} + \beta_{age} \text{ x age} + \beta_{BMI} \text{ x BMI} + \beta_{smoking} \text{ x Smoking status} + \beta_{Neu} \text{ x Neu} + \beta_{Eos} \text{ x Eos} + \beta_{Mono} \text{ x Mono} + \beta_{arrayrow} \text{ x array row} + \beta_{sampleplate2} \text{ x sample plate 2… + …} \beta_{sampleplate\ N} \text{ x sample plate N} + \varepsilon$$

For DNA methylation in buccal cells in children

$$CpGi = \alpha + \beta_{handedness} \text{ x left-handedness} + \beta_{sex} \text{ x sex} + \beta_{age} \text{ x age} + \beta_{gestational\ age} \text{ x gestational age} + \beta_{birth\ weight} \text{ x Birth Weight} + \beta_{maternal\ smoking} \text{ x Maternal Smoking} + \beta_{Epi} \text{ x Epi} + \beta_{NK} \text{ x NK} + \beta_{arrayrow} \text{ x array row} + \beta_{sampleplate2} \text{ x sample plate 2… + …} \beta_{sampleplate\ N} \text{ x sample plate N} + \varepsilon$$

ALSPAC

For DNA methylation in peripheral blood in adults (from 16 years old)

$$CpGi = \alpha + \beta_{handedness} \text{ x left-handedness} + \beta_{sex} \text{ x sex} + \beta_{age} \text{ x age} + \beta_{BMI} \text{ x BMI} + \beta_{smoking} \text{ x Smoking status} + \beta_{B\ lym} \text{ x B lym} + \beta_{CD4T} \text{ x CD4T} + \beta_{CD8T} \text{x CD8T} + \beta_{NK} \text{ x NK} + \beta_{NK} \text{ x NK} + \beta_{Mono} \text{ x Mono} + \beta_{Gran} \text{ x Gran} + \beta_{surrogate\ variable2} \text{ x surrogate variable 2 +….} \beta_{surrogate\ variableN} \text{ x surrogate variable N} + \varepsilon$$

For DNA methylation in cord blood

$$CpGi = \alpha + \beta_{handedness} \text{ x left-handedness} + \beta_{sex} \text{ x sex} + \beta_{gestational\ age} \text{ x gestational age} + \beta_{birth\ weight} \text{ x Birth Weight} + \beta_{maternal\ smoking} \text{ x Maternal Smoking} + \beta_{B\ lym} \text{ x B lym} + \beta_{CD4T} \text{ x CD4T} + \beta_{CD8T} \text{x CD8T} + \beta_{NK} \text{ x NK} + \beta_{NK} \text{ x NK} + \beta_{Mono} \text{ x Mono} + \beta_{Gran} \text{ x Gran} + \beta_{nRBC} \text{ x nRBC} + \beta_{surrogate\ variable2} \text{ x surrogate variable 2 +….} \beta_{surrogate\ variableN} \text{ x surrogate variable N} + \varepsilon$$

For DNA methylation in peripheral blood in children

$$CpGi = \alpha + \beta_{handedness} \text{ x left-handedness} + \beta_{sex} \text{ x sex} + \beta_{age} \text{ x age} + \beta_{gestational\ age} \text{ x gestational age} + \beta_{birth\ weight} \text{ x Birth Weight} + \beta_{maternal\ smoking} \text{ x Maternal Smoking} + \beta_{B\ lym} \text{ x B lym} + \beta_{CD4T} \text{ x CD4T} + \beta_{CD8T} \text{x CD8T} + \beta_{NK} \text{ x NK} +$$

$\beta_{Gran} \times Gran + \beta_{NK} \times NK + \beta_{Mono} \times Mono + \beta_{nRBC} \times nRBC + \beta_{surrogate\ variable2} \times surrogate\ variable\ 2 + \ldots.\ \beta_{surrogate\ variableN} \times surrogate\ variable\ N + \varepsilon$

where CpGi = DNA methylation β-value at methylation site i, α = the intercept, *left-handedness* is coded as 1=left-handed, and 0=right-handed; sex is coded 0 for males and 1 for females, age = the age at DNA methylation measurement in years, BMI = body mass index, smoking = smoking status (0=no, 1=former smoking, 2=current smoking), maternal smoking (0=not smoked, 1=smoked), Mon = percentage of monocytes, Eos = percentage of eosinophils, Neu = percentage of neutrophils, Blym = percentage of B lymphocytes, CD4T = percentage of CD4 + T-lymphocytes, CD8T = percentage of CD8 + T-lymphocytes, NK = percentage/proportion of natural killer cells, Gran = percentage of granulocytes, nRBC=nucleated red blood cells, Epi = percentage/proportion of epithelial cells, arrow row = the row of the sample on the Illumina 450k (ranging from 1 to 6) or EPIC Beadchip (ranging from 1 to 8), sample plate = bisulfite plate (dummy-coding) in NTR, surrogate variable in ALSPAC (n=20), and ε is residual.

Secondary analysis

GWAS follow-up

Differences between t-statistics of CpGs located in a 1 Mb window of SNPs derived from the GWAS of handedness and all other CpGs were tested with a linear regression model:

$t = \alpha + \beta_{handCpGs\_w1Mb} \times handCpGs\_w1Mb + \varepsilon$

where t = t-statistic, α = the intercept, handCpGs_w1Mb = variable indicating if the CpG was located in a 1 Mb window of SNPs derived from the GWAS of handedness (0=no/1=yes)

Polygenic and methylation scores testing

We tested whether a methylation score (MS) adds predictive value for handedness over and above the polygenic score (PGS), and calculated the variance in handedness that is explained by the PGS and MS. We calculated the variance explained on the liability scale (Lee et al, 2012[1]). To this end we ran five logistic regression models with the R function *glm*:

Prediction by PGS:

Model 1: PGS and GWAS covariates*

$left\text{-}handedness = \alpha + \beta_{PGS} \times PGS + \beta_{age} \times age + GWAS\ covariates$

Model 2: genotype covariates

$left\text{-}handedness = \alpha + \beta_{age} \times age + GWAS\ covariates$

Prediction by PGS and MS:

Model 3: PGS, MS, and GWAS and EWAS covariates**

$left\text{-}handedness = \alpha + \beta_{PGS} \times PGS + \beta_{age} \times age + \beta_{sex} \times sex + \beta_{PGS} \times MS + GWAS\ covariates + EWAS\ covariates$

Model 4: with PGS, GWAS and EWAS covariates

$left\text{-}handedness = \alpha + \beta_{PGS} \times PGS + \beta_{age} \times age + \beta_{sex} \times sex + GWAS\ covariates + EWAS\ covariates$

Model 5: with MS, GWAS and EWAS covariates

$handedness = \alpha + \beta_{PGS} \times MS + \beta_{age} \times age + \beta_{sex} \times sex + GWAS\ covariates + EWAS\ covariates$

* GWAS covariates included dummy variables for platforms and 10 principal components based on genotype data in NTR and 10 principal components in ALSPAC.

** EWAS covariates included BMI, smoking (for adults), gestational age, birth weight, maternal smoking (for children), percentage/proportions of cells, bisulfite plate (dummy-coding) in NTR, surrogate variable in ALSPAC (n=20).

Model 3 was run for three MS with different sets of CpGs included by varying the p-value threshold (p-value $<1\times10^{-1}$, $<1\times10^{-3}$, $<1\times10^{-5}$).

Calculation of R^2 (based on Lee et al[1]). R^2 is equal to the explained variance divided by the total variance; that is the sum of explained variance and residual (homoscedastic) variance. We first regressed left-handedness on PGS and GWAS covariates (genotype platform, the first ten principal components based on genotype data, and sex) (model 1), and then on GWAS covariates only (model 2) using logistic regression. We calculated variance explained by all predictors in each model. We calculated the predictive value of the PGS by subtracting the difference between the variance explained by the first and the second model. For BMI, it has been shown that DNA methylation predicts the trait over and above a polygenic score based on SNPs[2]. To examine the predictive value of MS and PGS in a combined model, we regressed left-handedness on PGS, MS, genotyping and EWAS covariates (model 3). Next, we regressed left-handedness on the same predictors without MS (model 4) and without PGS (model 5). The difference between explained variance in the third and fourth models gave us an estimate of variance explained by MS. The difference between explained variance of the third and fifth models resulted in an estimate explained by PGS.

Residual (homoskedastic) variance:

$$\text{Res.Var} = \pi^2 / 3$$

Explained variance whole model:

$$\text{Ex.Var} = \text{var}(\beta_1 * \text{predictor}_1 + \beta_2 * \text{predictor}_2 + \ldots + \beta N * \text{predictorN})$$

Proportion of explained variance in total variance:

$$R^2 = \text{Ex.Var} / (\text{Ex.Var} + \text{Res.Var})$$

where β_N=regression coefficient of the Nth predictor in the model.

Explained variance for PGS:

Exp.Var. PGS = Total Exp.Var Model 1 – Total Exp.Var.Model 2

Explained variance for PGS in the combined model:

Exp.Var. PGS = Total Exp.Var Model 3 – Total Exp.Var.Model 5

Explained variance for MS in the combined model:

Exp.Var. MS = Total Exp.Var Model 3 – Total Exp.Var.Model 4

Reference

1. Lee, S. H., Goddard, M. E., Wray, N. R. & Visscher, P. M. A better coefficient of determination for genetic profile analysis. Genet. Epidemiol. 36, 214–224 (2012).
2. Shah, S. et al. Improving Phenotypic Prediction by Combining Genetic and Epigenetic Associations. Am. J. Hum. Genet. 97, 75–85 (2015).

Supplementary Table 1. Primary analysis group characteristics

	NTR adults					ALSPAC adults				
	N	total	LH	RH	*P*	*N*	total	LH	RH	*P*
N (%)		2682	324 (12%)	2358 (88%)			1232	99 (8%)	1133 (92%)	
Age at blood sampling (years), mean (SD)	2682	36.5(12.7)	34.3(11.2)	36.8(12.9)	0.001	1232	48.98 (5.55)	49.08 (5.92)	48.98 (5.51)	0.861
Age range		[17.6-79.6]	[17.8-79.6]	[17.6-79.2]			[31-75]	[31-70]	[32.9-75]	
Sex	2682					1232				
Males, n (%)		902 (34%)	119 (37%)	783 (33%)	0.208		364 (29.5%)	31 (31.3%)	333 (29.4%)	
Females, n (%)		1780 (66%)	205 (63%)	1575 (67%)			868 (70.5%)	68 (68.7%)	800 (70.7%)	
BMI, mean (SD)	2667	24.2 (3.9)	24.2 (3.7)	24.2 (3.9)	0.977	1095	26.6 (4.7)	25.8 (4.25)	26.7 (4.74)	0.099
Current smoking, *n (%)*	2677	551 (20.6%)	65 (20.2%)	486 (20.6%)	0.964	1232	461 (37.4%)	37 (37.4%)	424 (37.4%)	0.101
Cell percentage, mean (SD)			*Neutrophils*					*B lymphocytes*		
	2682	52.4 (9.1)	52.8 (8.7)	52.4 (9.2)	0.485	1232	10.42 (4.03)	10.76 (4.3)	10.39 (4)	0.38
			Eosinophils					*CD4T*		
	2682	3.09(2.23)	3.1 (1.89)	3.1 (2.27)	0.945	1232	18.1 (6.69)	18.4 (7.23)	18.0 (6.64)	0.601
			Monocytes					*CD8T*		
	2682	8.4 (2.3)	8.4 (2.16)	8.4 (2.4)	0.922	1232	1.87 (3.18)	2.16 (3.77)	1.85 (3.13)	0.351
**Twin-specific characteristics**								*Natural killer cells*		
Singletons, *n (%)*		196 (7.3%)	9 (2.7%)	187 (8%)		1232	20.8 (5.81)	21.95 (6.51)	20.7 (5.73)	0.038
Multiples, *n (%)*		2486 (92.7%)	315 (97.2%)	2171 (92%)				*Granulocytes*		
Zygosity	2484					1232	47.7 (12.61)	45.5 (14.72)	47.9 (12.4)	0.073
MZ, n (%)		1542 (62%)	190 (60.3%)	1352 (62.3%)	0.489			*Monocytes*		
DZ, n (%)		942 (37.9%)	125 (39.7%)	817 (37.7%)		1232	7.43 (3.49)	7.5 (3.36)	7.43 (3.5)	0.855
Handedness discordance in MZ										
Discordant, n (%)		266 (20.8%)	133 (81.1%)	133 (11.9%)						
Concordant LH, n (%)		31 (2.4%)	31 (18.9%)	-						
Concordant RH, n (%)		982 (76.8%)	-	982 (88%)						

NTR, Netherlands Twin Register. ALSPAC, Avon Longitudinal Study of Parents and Children. LH, left-handed. RH, right-handed. SD, standard deviation. *P*, *P*-value for intragroup differences between LH and RH. Percentage for LH and RH in N(%) is by row, in others percentages are by column.

Supplementary Table 2. Year of birth and handedness in NTR adults

		<=1939	1940-49	1950-59	1960-69	1970-79	1980-89	1990+	total
Left-handed	Count	4	12	35	35	181	61	10	338
	%	4.2%	5.9%	11.1%	10.2%	13.4%	13.4%	13.7%	11.9%
Right-handed	Count	79	186	267	298	1147	390	63	2430
	%	82.3%	90.7%	84.5%	86.9%	84.6%	85.7%	86.3%	85.5%
Ambidextrous	Count	13	7	14	10	27	4	0	75
	%	13.5%	3.4%	4.4%	2.9%	2.0%	0.9%	0.0%	2.6%
	Count	96	205	316	343	1355	455	73	2843

Supplementary Table 3. Secondary analysis group characteristics

	NTR children (buccal cells)					ALSPAC at birth (cord blood)					ALSPAC 7 years old (blood)					ALSPAC 17 years old (blood)					ALSPAC 24 years old (blood)				
	N	total	LH	RH	*P**	*N*	total	LH	RH	*P*	*N*	total	LH	RH	*P*	*N*	total	LH	RH	*P*	*N*	total	LH	RH	*P*
N		946	139 (15%)	807 (85%)			703	60 (8.5%)	643 (91.5%)			757	68 (9%)	689 (91%)			759	69 (9.1%)	690 (90.9%)			442	37 (8.4%)	405 (91.6%)	
Age at blood sampling (mean (SD))	946	9.57 (1.85)	9.58 (1.78)	9.56 (1.86)	0.943						757	7.44 (0.13)	7.43 (0.08)	7.44 (0.13)	0.585	759	17.11 (1.04)	16.91 (1.13)	17.13 (1.03)	0.082	442	24.33 (0.74)	24.3 (0.67)	24.33 (0.74)	0.76
Sex	946					703					757														
Males, n(%)		483 (51%)	71 (51%)	412 (51%)	0.999		334 (47.5%)	32 (53.3%)	302 (47%)	0.348		368 (48.6%)	36 (52.9%)	332 (48.2%)	0.525	759	357 (47%)	36 (52.2%)	321 (46.5%)	0.379	442	189 (57.2%)	16 (43.2%)	173 (42.7%)	1
Females, n(%)		463 (49%)	68 (49%)	395 (49%)			369 (52.5%)	28 (46.7%)	341 (53%)			389 (51.4%)	32 (47.1%)	357 (51.8%)			402 (53%)	33 (47.8%)	369 (53.5%)			253 (57.2%)	21 (56.8%)	232 (57.3%)	
Gestational age, mean (SD)	918	35.88 (2.57)	35.51 (2.83)	35.93 (2.52)	0.298	703	39.6 (1.53)	39.63 (1.37)	39.57 (1.55)	0.763	756	39.6 (1.53)	39.6 (1.37)	39.6 (1.55)	0.783										
Birth weight (children), BMI (>=16 years old), mean (SD)	914	2401 (541.2)	2369 (585.2)	2407 (533.5)	0.407	694	3485 (489.2)	3567.8 (434.4)	3477.3 (493.6)	0.174	747	3490.4 (489.8)	3582.9 (445.4)	3481.3 (493.3)	0.105	650	22.47 (3.64)	21.87 (3.76)	22.53 (3.63)	0.191	439	24.35 (4.46)	24.22 (3.55)	24.36 (4.54)	0.847
Maternal smoking during pregnancy (yes), n(%)	874	70 (8%)	14 (11%)	56 (7%)	0.189	697	83 (11.9%)	9 (15.2%)	74 (11.6%)	0.401	752	91 (12.1%)	10 (14.9%)	81 (11.8%)	0.435										
Cell proportions, mean (SD)			*Epithelium cells*					*B lymphocytes*					*B lymphocytes*					*B lymphocytes*					*B lymphocytes*		
	946	0.81 (0.112)	0.78 (0.14)	0.82 (0.1)	0.001	703	0.17 (0.04)	0.18 (0.05)	0.17 (0.04)	0.488	757	0.14 (0.03)	0.14 (0.03)	0.14 (0.03)	0.24	759	0.11 (0.03)	0.11 (0.03)	0.11 (0.03)	0.477	442	0.11 (0.02)	0.12 (0.02)	0.11 (0.02)	0.398
			Natural killer cells					*CD4T*					*CD4T*					*CD4T*					*CD4T*		
	946	0.03 (0.012)	0.032 (0.013)	0.029 (0.012)	0.013	703	0.18 (0.06)	0.19 (0.06)	0.18 (0.06)	0.553	757	0.21 (0.05)	0.21 (0.05)	0.21 (0.05)	0.81	759	0.18 (0.05)	0.18 (0.05)	0.18 (0.05)	0.558	442	0.18 (0.06)	0.19 (0.05)	0.18 (0.06)	0.436
								CD8T					*CD8T*					*CD8T*					*CD8T*		
**Twin-specific characteristics**						703	0.1 (0.05)	0.10 (0.04)	0.10 (0.05)	0.629	757	0.04 (0.04)	0.03 (0.04)	0.04 (0.04)	0.231	759	0.03 (0.04)	0.03 (0.03)	0.03 (0.04)	0.503	442	0.02 (0.03)	0.02 (0.03)	0.02 (0.03)	0.249
Zygosity	946				0.298			*Natural killer cells*					*Natural killer cells*					*Natural killer cells*					*Natural killer cells*		
MZ n(%)		794 (82%)	121 (87%)	673 (84%)		703	0.01 (0.02)	0.01 (0.01)	0.01 (0.02)	0.18	757	0.19 (0.05)	0.20 (0.04)	0.19 (0.05)	0.135	759	0.21 (0.06)	0.22 (0.06)	0.21 (0.06)	0.428	442	0.21 (0.05)	0.22 (0.05)	0.21 (0.05)	0.897
DZ n(%)		152 (18%)	18 (13%)	134 (16%)				*Granulocytes*					*Granulocytes*					*Granulocytes*					*Granulocytes*		
Handedness discordance in MZ						703	0.35 (0.1)	0.34 (0.10)	0.35 (0.10)	0.51	757	0.44 (0.08)	0.44 (0.07)	0.44 (0.08)	0.968	759	0.47 (0.09)	0.47 (0.08)	0.47 (0.09)	0.82	442	0.49 (0.09)	0.47 (0.09)	0.49 (0.09)	0.351
Discordant n(%)		172 (24%)	86 (83%)	86 (14%)				*Monocytes*					*Monocytes*					*Monocytes*					*Monocytes*		
Concordant LH n(%)		18 (3%)	18 (17%)	-		703	0.01 (0.02)	0.01 (0.01)	0.01 (0.02)	0.18	757	0.06 (0.03)	0.06 (0.03)	0.06 (0.03)	0.179	759	0.06 (0.03)	0.06 (0.03)	0.06 (0.03)	0.109	442	0.05 (0.03)	0.05 (0.03)	0.05 (0.03)	0.717
Concordant RH n(%)		520 (73%)	-	520 (85%)				*nRBC*																	
						703	0.19 (0.09)	0.18 (0.09)	0.19 (0.09)	0.5															

NTR, Netherlands Twin Register. ALSPAC, Avon Longitudinal Study of Parents and Children. LH, left-handed. RH, right-handed. SD, standard deviation. nRBC, nucleated red blood cells. *P*, P-value for differences between LH and RH. *P-values from GEE (corrected for relatedness). Percentage for LH and RH in *n* (%) by row, in others percentage is by column

Supplementary Table 4. Characteristics of discordant MZ twins

	LH		RH	*P*
NTR Adults, N	133		133	
Age at blood sampling, mean (SD)	32.8(9.7)		32.7(9.7)	
Sex in each group				
Males, n (%)		39 (29%)		
Females, n (%)		94 (71%)		
BMI, mean (SD)	24.2(4.2)		24.3(3.9)	0.35
Current smoking, *n (%)*	25(19%)		21(16%)	0.646
Neutrophils percentage, mean (SD)	52.73(8.97)		53.54(9.75)	0.975
Eosinophils percentage, mean (SD)	2.89(1.72)		2.81(2.01)	0.889
Monocytes percentage, mean (SD)	8.56(2.09)		8.38(2.24)	0.906
NTR Children, N	86		86	
Age at buccal cells sampling (mean (SD))	9.8(1.81)		9.8(1.81)	
Sex in each group				
Males, n (%)		45(52%)		
Females, n (%)		41(48%)		
Gestational age, mean (SD)		35(3.15)		
Prenatal maternal smoking, *n (%)*		14(9%)		
Birth weight, mean (SD)	2246.6(582.2)		2273.73(600.1)	0.762
Epithelium cells proportion in buccal swabs, mean (SD)	0.78(0.14)		0.79(0.118)	0.578
Natural killer cells proportion in buccal swabs, mean (SD)	0.031(0.012)		0.031(0.012)	0.666

NTR, Netherlands Twin Register. LH, left-handed. RH, right-handed. SD, standard deviation. *P*, *P*-value for differences between LH and RH discordant twins. Percentage is given by column.

PART 1. PRIMARY ANALYSIS

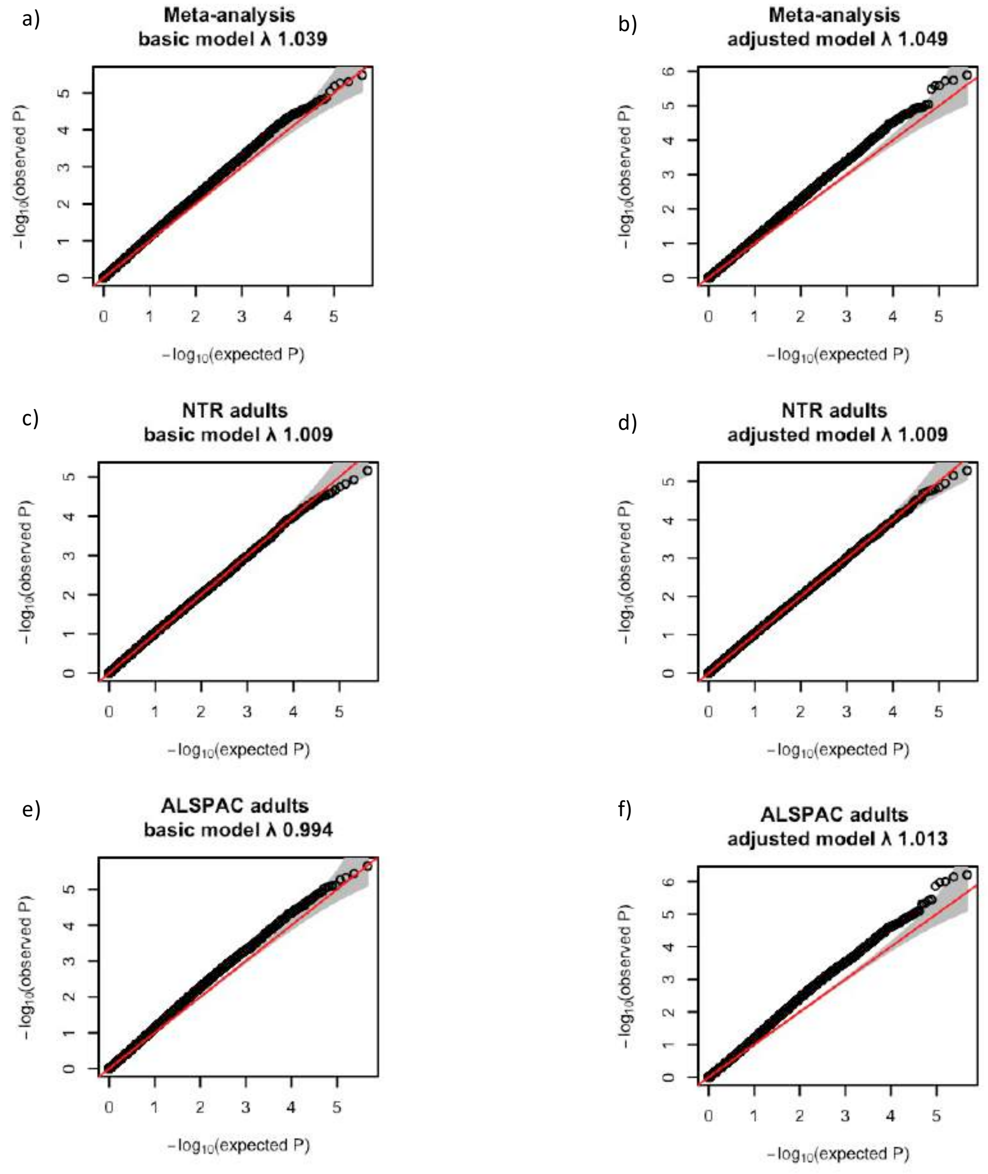

Supplementary Figure 1. QQ plots of EWAS results on left-handedness in primary analysis

Quantile-quantile (QQ) plot from the EWAS of left-handedness in different analyses: **a)** Meta-analysis (NTR adults and ALSPAC adults) basic model (*N*=3941); **b)** Meta-analysis (NTR adults and ALSPAC adults) adjusted model (*N*=3721); **c)** NTR adults, basic model (*N*=2710); **d)** NTR adults, adjusted model (*N*=2663); **e)** ALSPAC adults, basic model (*N*=1232); **f)** ALSPAC adults, adjusted model (*N*=1058)
The observed p-values (y-axis) are plotted against the p-values expected under the null hypothesis (x-axis). The straight diagonal line denotes the pattern expected under the null hypothesis, with 95% confidence intervals indicated by the shaded grey line. λ, Bayesian estimate of inflation.

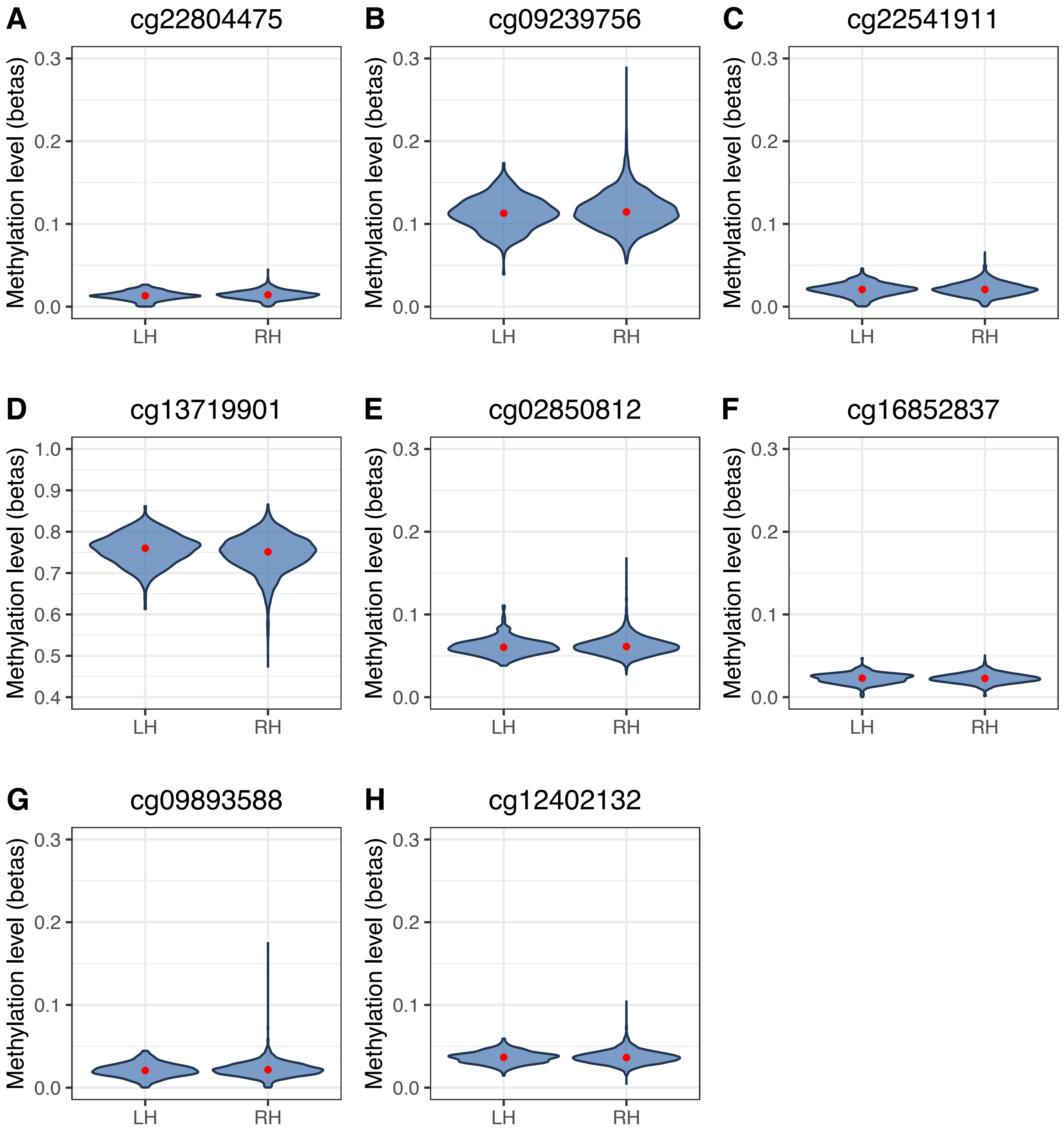

Supplementary Figure 2. DNA methylation level at top CpGs from meta-analysis in right- and left-handed in NTR

The plots depict the mean DNA methylation level in blood (Illumina 450k) in left-handers (LH) and right-handers (RH) represented by red dot for top CpGs from meta-analysis adjusted model at $P < 1.0 \times 10^{-5}$ ($N_{\text{NTR adults}} = 2663$).

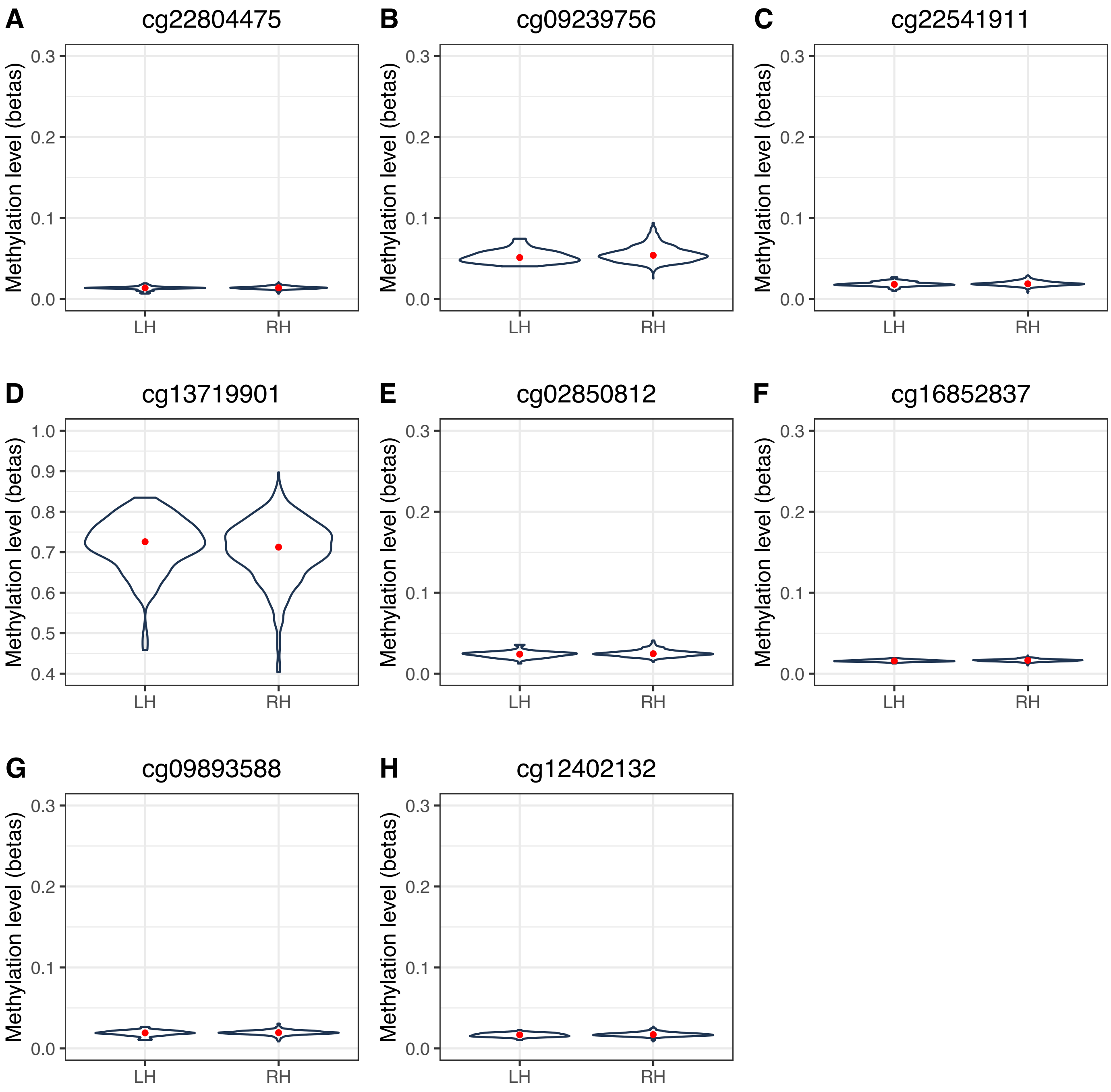

Supplementary Figure 3. DNA methylation level at top CpGs from meta-analysis in right- and left-handed in ALSPAC

The plots depict the mean DNA methylation level in blood (Illumina 450k) in left-handers (LH) and right-handers (RH) represented by red dot for top CpGs from meta-analysis adjusted model at $P < 1.0 \times 10^{-5}$ ($N_{\text{ALSPAC adults}} = 1058$).

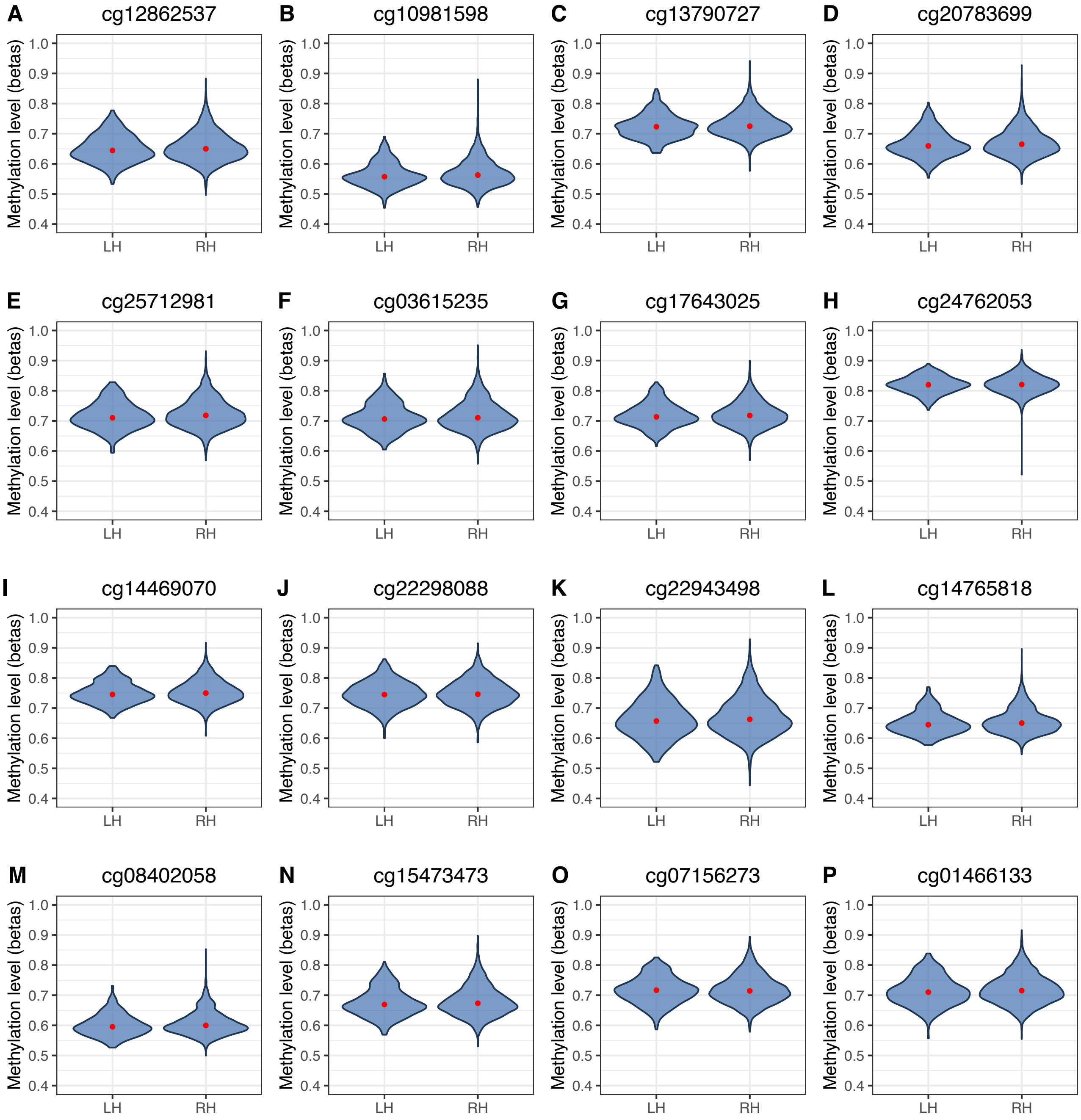

Supplementary Figure 4. DNA methylation level at CpGs from left-handedness associated DMR at chromosome 20 in right- and left-handed in NTR

The plots depict the mean DNA methylation level in blood (Illumina 450k) for each CpGs in DMR at chromosome 20 in left-handers (LH) and right-handers (RH) represented by red dot ($N_{NTR\ adults}$ = 2663).

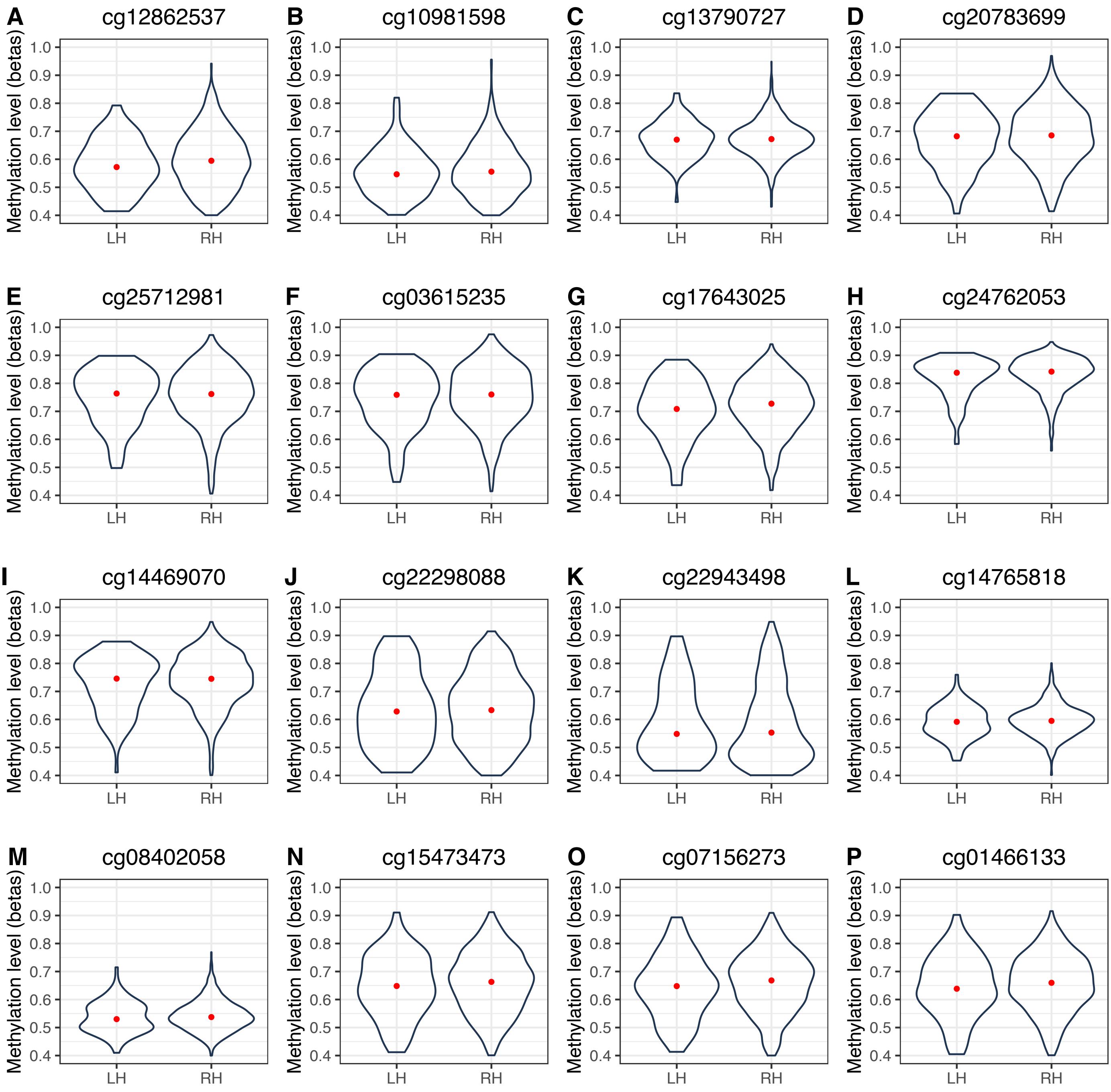

Supplementary Figure 5. DNA methylation level at CpGs from left-handedness associated DMR at chromosome 20 in right- and left-handed in ALSPAC

The plots depict the mean DNA methylation level in blood (Illumina 450k) for each CpGs in DMR at chromosome 20 in left-handers (LH) and right-handers (RH) represented by red dot ($N_{\text{ALSPAC adults}}$ = 1058).

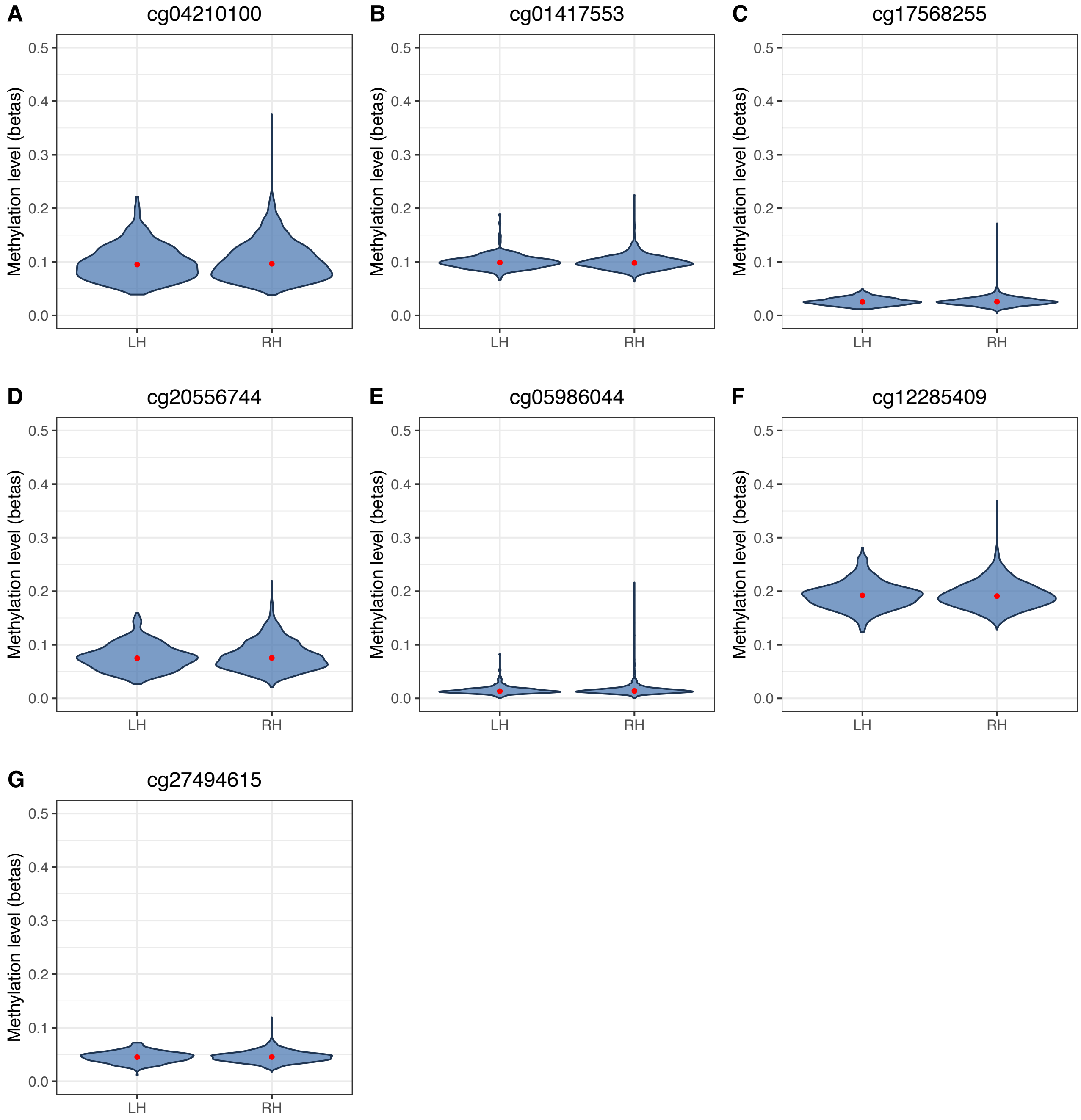

Supplementary Figure 6. DNA methylation level at CpGs from left-handedness associated DMR at chromosome 2 in right- and left-handed in NTR

The plots depict the mean DNA methylation level in blood (Illumina 450K) for each CpGs in DMR at chromosome 2 in left-handers (LH) and right-handers (RH) represented by red dot ($N_{NTR\ adults}$ = 2663).

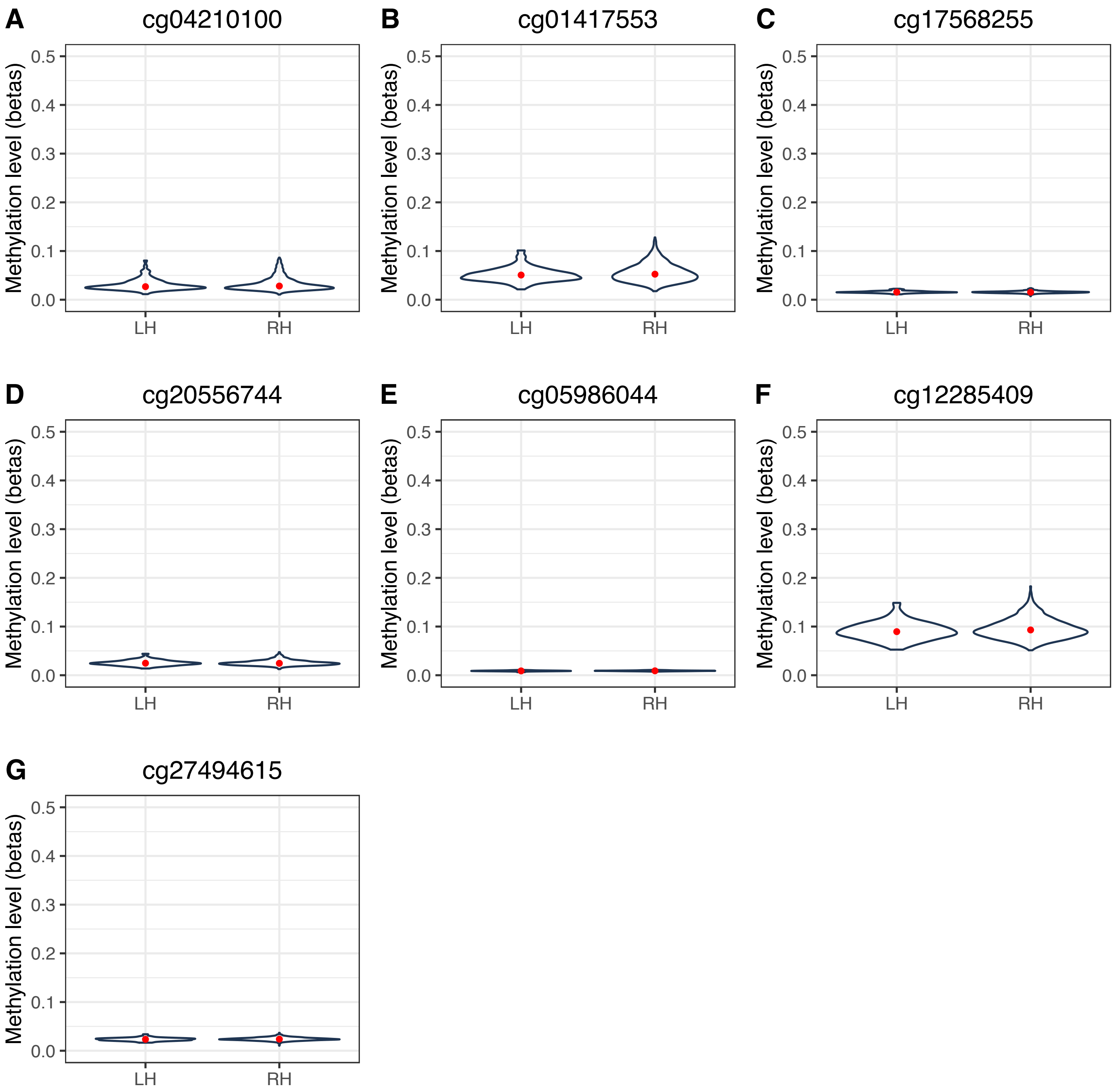

Supplementary Figure 7. DNA methylation level at CpGs from left-handedness associated DMR at chromosome 2 in right- and left-handed in ALSPAC

The plots depict the mean DNA methylation level in blood (Illumina 450K) for each CpGs in DMR at chromosome 2 in left-handers (LH) and right-handers (RH) represented by red dot ($N_{\text{ALSPAC adults}}$ = 1058).

Supplementary Table 14. GWAS follow-up results

SNP fraction	N SNPs	N CpGs located within 1Mb window	*ß*	Bootstrap SE	Bootstrap *P*
LH p<5x10^{-08}	420	2,784	0.027	0.013	0.039
LH p<5x10^{-08}	420	2,567 *	0.027	0.013	0.027
LH p<1x10^{-06}	2,625	14,631	0.011	0.006	0.048
LH p<1x10^{-05}	3,464	35,975	0.003	0.004	0.472
T2D p<5x10^{-08}	2,392	27,229	0.005	0.004	0.265

Note: Results of linear regression of absolute z-scores of CpGs on a variable (yes/no) indicating if CpGs were located within 1Mb from SNPs associated with the trait. The analysis was performed using EWAS summary statistics from the meta-analysis. GWAS summary statistics were obtained for left-handedness from Cuellar-Partida et al. (2020) and for type 2 diabetes from Watanabe et al, 2019 (available at GWAS atlas https://atlas.ctglab.nl/traitDB/3686; 41204_E11_logistic.EUR.sumstats.MACfilt.txt).
LH, left-handedness; T2D, type 2 diabetes.; ß, regression coefficient; Bootstrap SE, standard error computed with bootstraps; Bootstrap *P*, *P*-value computed with bootstrap SE.
* CpGs driven by mQTL removed

a) b)

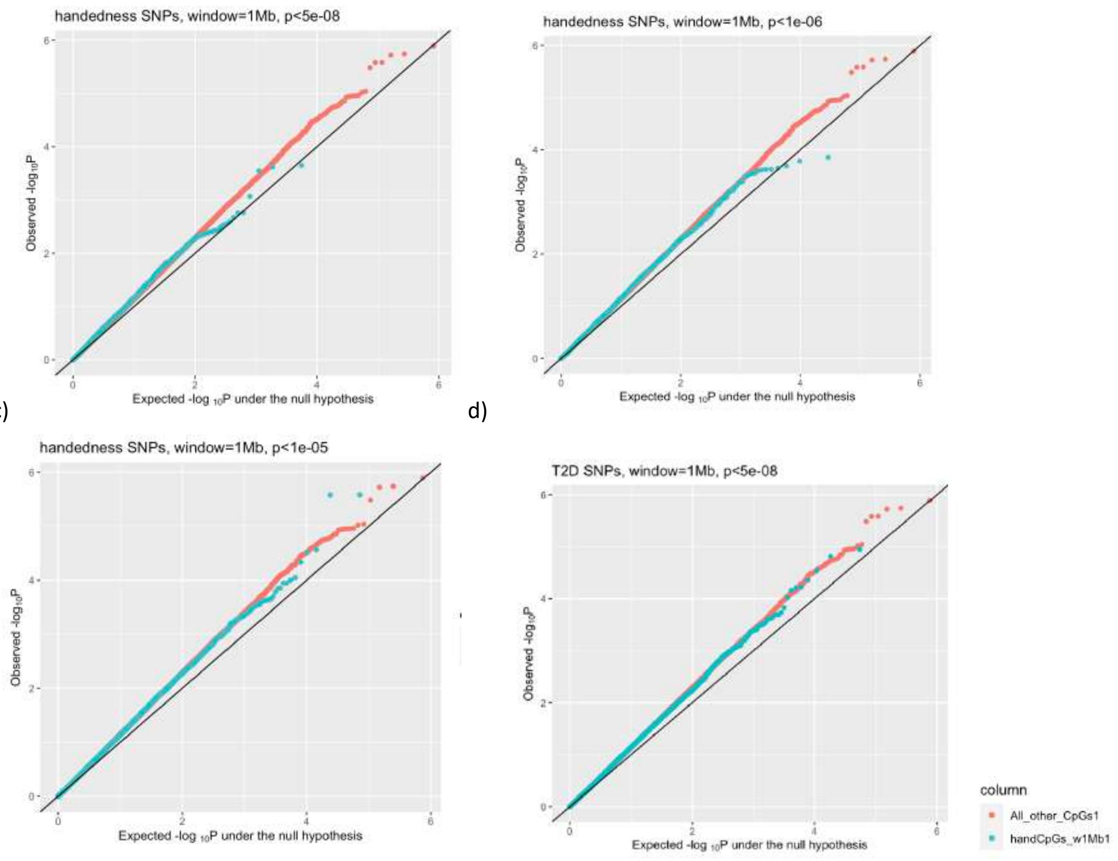

Supplementary Figure 8. QQ plots of p-values of CpGs located within 1Mb window from GWAS SNPs associated with left-handedness and type 2 diabetes

a) QQ-plot of p-values of CpGs located within 1Mb window from handedness GWAS SNPs associated with handedness at *P* <5x10^{-08}; b) QQ-plot of *P*-values of CpGs located within 1Mb window from handedness GWAS SNPs associated with handedness at *P* <1x10^{-06}; c) QQ-plot of *P* -values of CpGs located within 1Mb window from handedness GWAS SNPs associated with handedness at *P* <1x10^{-05}; d) QQ-plot of *P*-values of CpGs located within 1Mb window from T2D GWAS SNPs associated with handedness at *P*<5x10^{-08}

PART 2. SECONDARY ANALYSIS

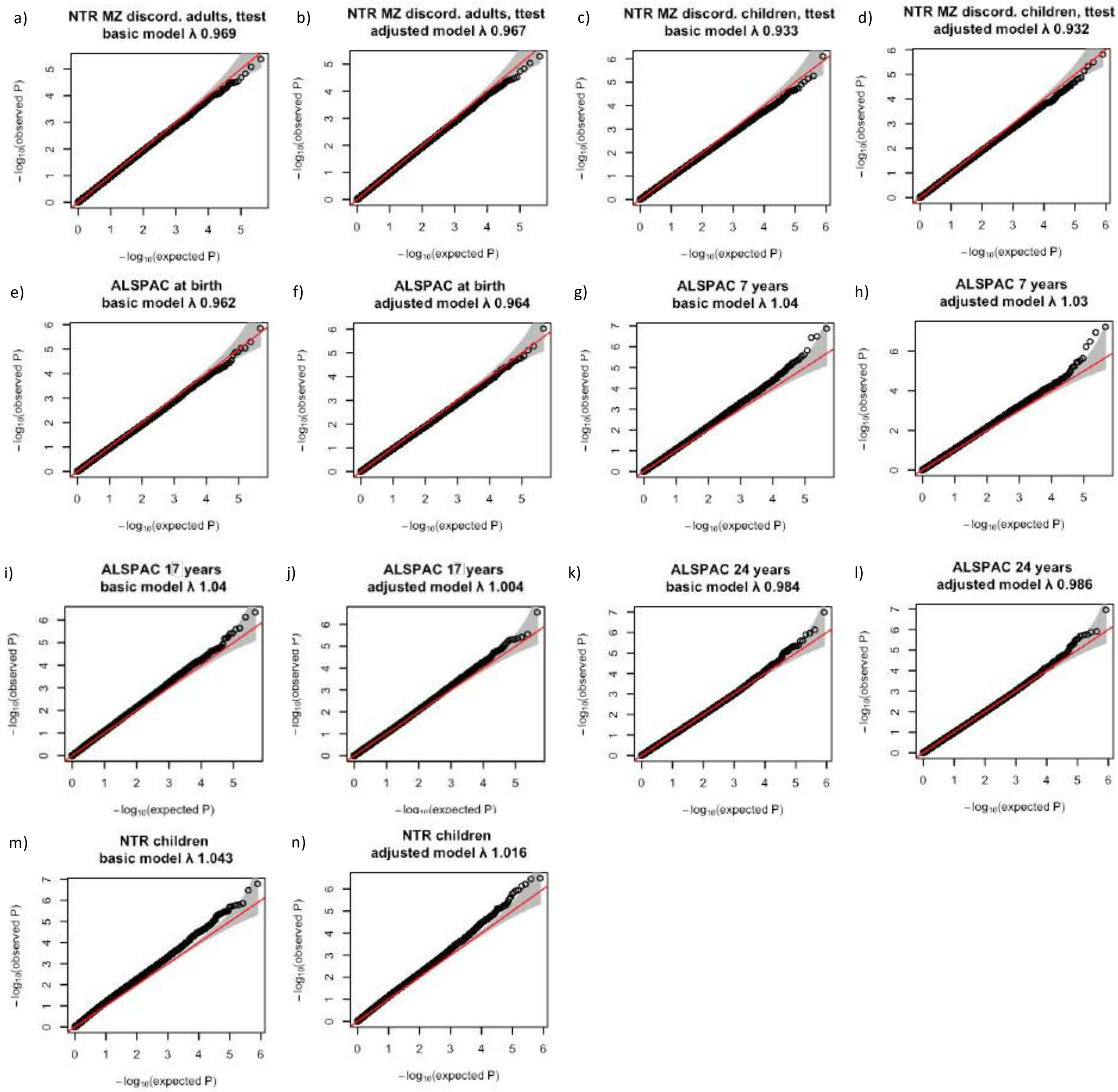

Supplementary Figure 9. QQ plots of EWAS results on left-handedness in secondary analysis

Quantile-quantile (QQ) plot from the EWAS of handedness in different analyses: **a)** MZ within-pair analysis, NTR adults, basic model (*N*=266); **b)** MZ within-pair analysis, NTR adults, adjusted model (*N*=264); **c)** MZ within-pair analysis, NTR children, basic model (*N*=172); **d)** MZ within-pair analysis, NTR children, adjusted model (*N*=168); **e)** ALSPAC offspring at birth, basic model (*N*=703); **f)** ALSPAC offspring at birth, adjusted model (*N*=688); **g)** ALSPAC offspring at 7 years old, basic model (*N*=757); **h)** ALSPAC offspring at 7 years old, adjusted model (*N*=742); **i)** ALSPAC offspring at 17 years old, basic model (*N*=759); **j)** ALSPAC offspring at 17 years old, adjusted model (*N*=641); **k)** ALSPAC offspring at 24 years old, basic model (*N*=442); **l)** ALSPAC at 24 years old, adjusted model (*N*=431); **m)** NTR children, basic model (*N*=946); **n)** NTR children, adjusted model (*N*=866).

The observed *P*-values (y-axis) are plotted against the p-values expected under the null hypothesis (x-axis). The straight diagonal line denotes the pattern expected under the null hypothesis, with 95% confidence intervals indicated by the shaded grey line. λ, Bayesian estimate of inflation

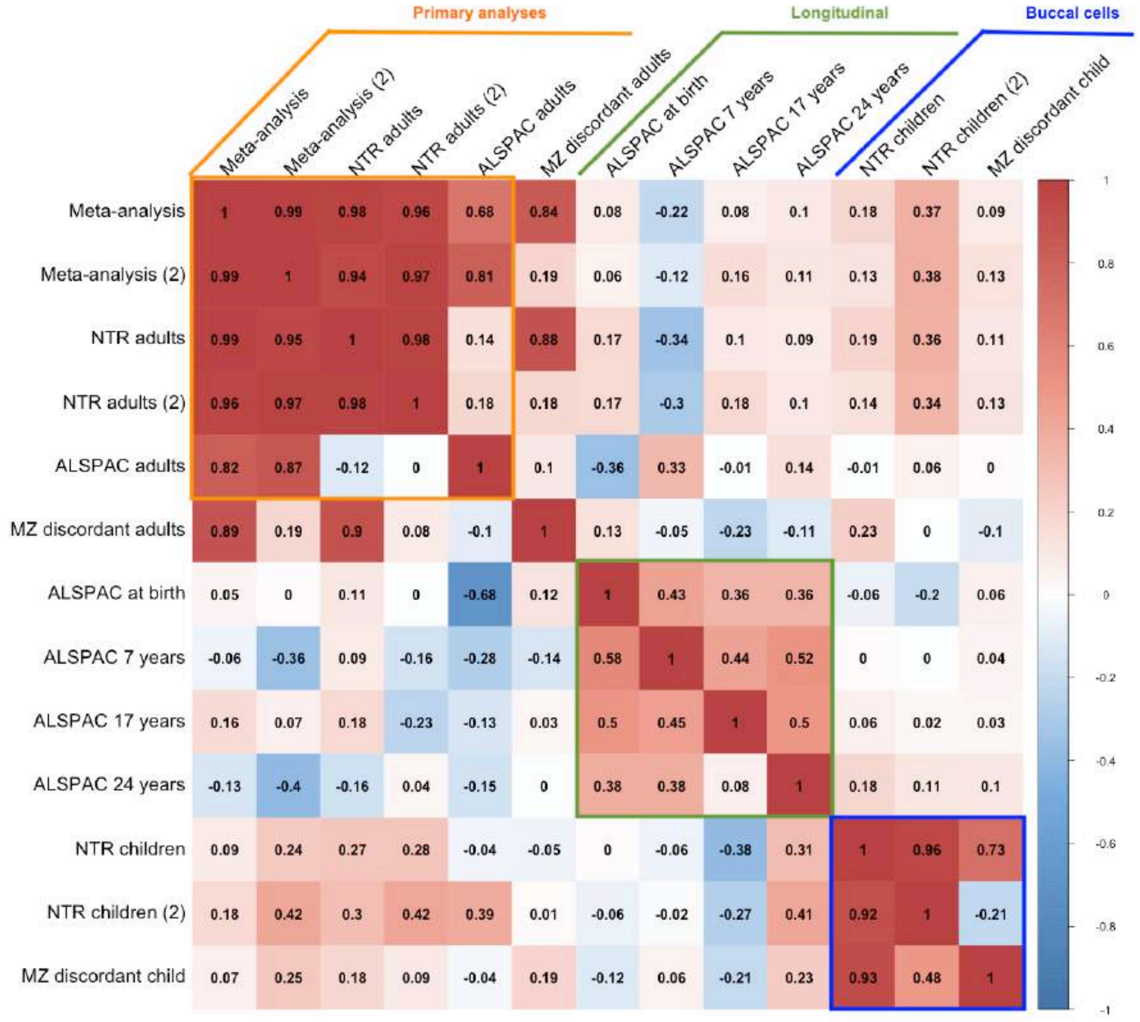

Supplementary Figure 10. Correlations among the effects of top 100 CpGs across analyses

Note: Orange frame = primary analysis. Green frame = longitudinal EWAS in ALSPAC at 4 points (at birth, 7 years, 17 years, 24 years). Blue frame = EWAS in NTR children (buccal cells). MZ discordant twins = MZ discordant twin within-pair EWAS. Meta-analysis (2), NTR adults (2), NTR children (2) = analyses without MZ discordant twins.

Correlation matrix is based on the 379,924 methylation sites available in all analyses (present on the EPIC array and 450k array). The lower triangle contains the correlations between effects (regression coefficients) of the top 100 CpGs ranked by p-value from the models listed on the horizontal axis with the effects of the same CpGs for the models listed on the vertical axis, and the upper triangle vice versa.

a) b)

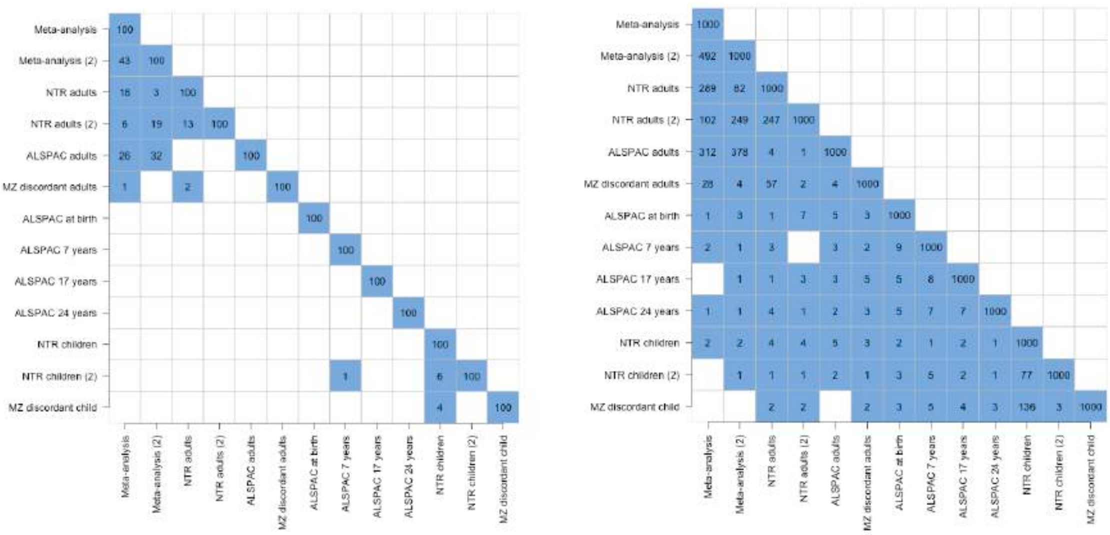

Supplementary Figure 11. Overlaps in top CpGs across analyses

a) top 100 CpGs; **b**) top 1000 CpGs. On the base of preselected list of 379,924 methylation sites available in all analyses. MZ discordant twins = MZ discordant twin within-pair EWAS. Meta-analysis (2), NTR adults (2), NTR children (2) = analyses without MZ discordant twins.

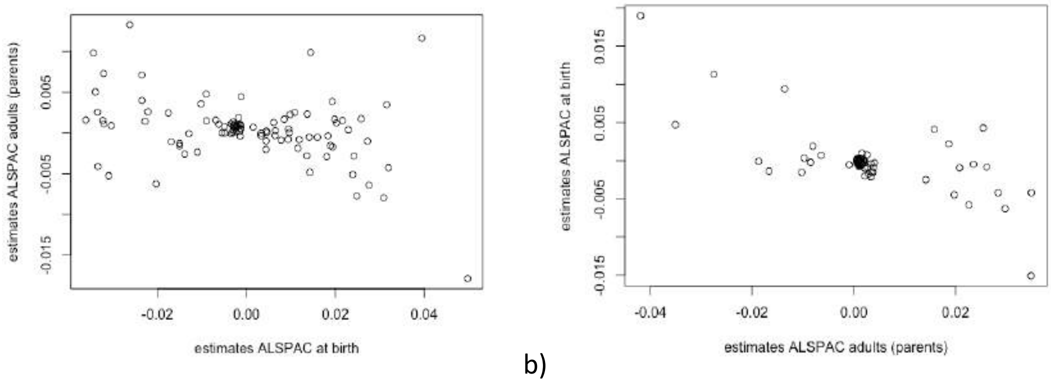

a) b)

Supplementary Figure 12. Scatterplot of effects in ALPAC adults and ALSPAC at birth

a) top 100 CpGs from ALSPAC offspring at birth EWAS, adjusted model (*N*=688), **b)** top 100 CpGs from ALSPAC adults (parents) EWAS, adjusted model (*N*=1058)

Note: Scatterplots were done to check if an outlier was the cause for unexpected negative correlation between top 100 estimates with lowest p-value $r^{ALSPACadults- ALSPACatbirth}$ =-0.680; *P*= 7.2 x 10^{-15}.

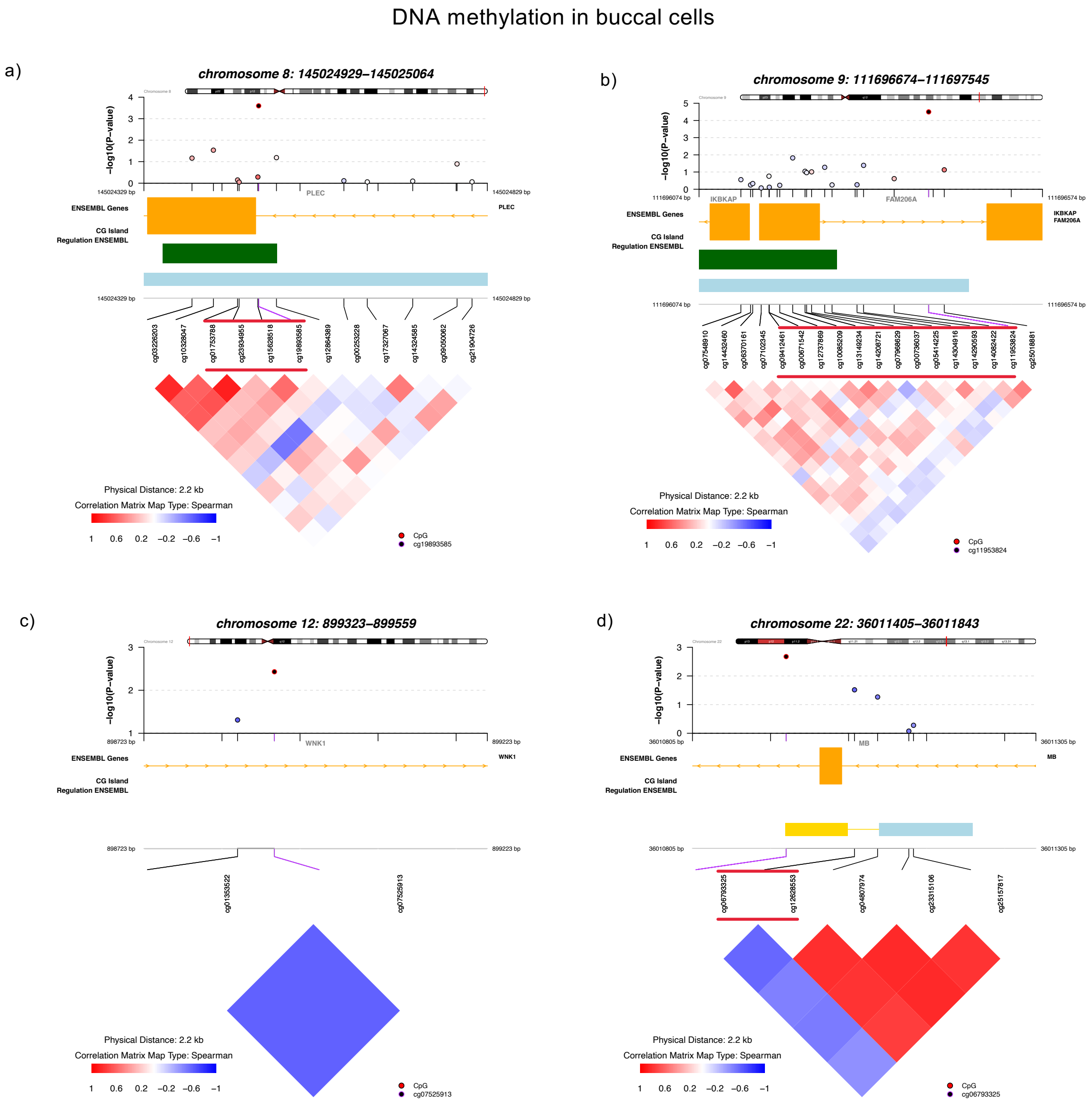

Supplementary Figure 13. Differentially methylated regions associated with left-handedness in secondary analysis

CpGs from DMRs are indicated with red lines. The top panel of each plot shows the EWAS p-values for all CpGs in the window, with the most strongly associated CpG highlighted. The middle panel shows the genomic coordinates (genome build GRCh37/hg19) and the functional annotation of the region: the ENSEMBL Genes track shows the genes in the genomic region (orange); the CpG Island track shows the location of CpG islands (green); the Regulation ENSEMBL track shows regulatory regions (blue). The bottom panel shows the Spearman correlation between methylation levels of CpGs in the window.
a) DMR at chromosome 8, EWAS, adjusted model, NTR children**; b)** DMR at chromosome 9, EWAS, adjusted model, NTR children; **c)** DMR at chromosome 12, EWAS, adjusted model, NTR children; **d)** DMR at chromosome 22, EWAS, adjusted model, NTR children.

Supplementary Table 30. Polygenic and methylation scores general description

	NTR adults	NTR children 9 years	ALSPAC mothers	ALSPAC children 7 years
PGS 0.5 fraction	6,452,863		6,779,197	
MS p < 1x10-1	66,712	48,125	43,628	46,698
MS p < 1x10-3	1536	5,041	413	546
MS p < 1x10-5	14	12	2	7

PGS, polygenic score; MS, methylation score. The numbers of CpGs/SNPs included in the scores are reported.

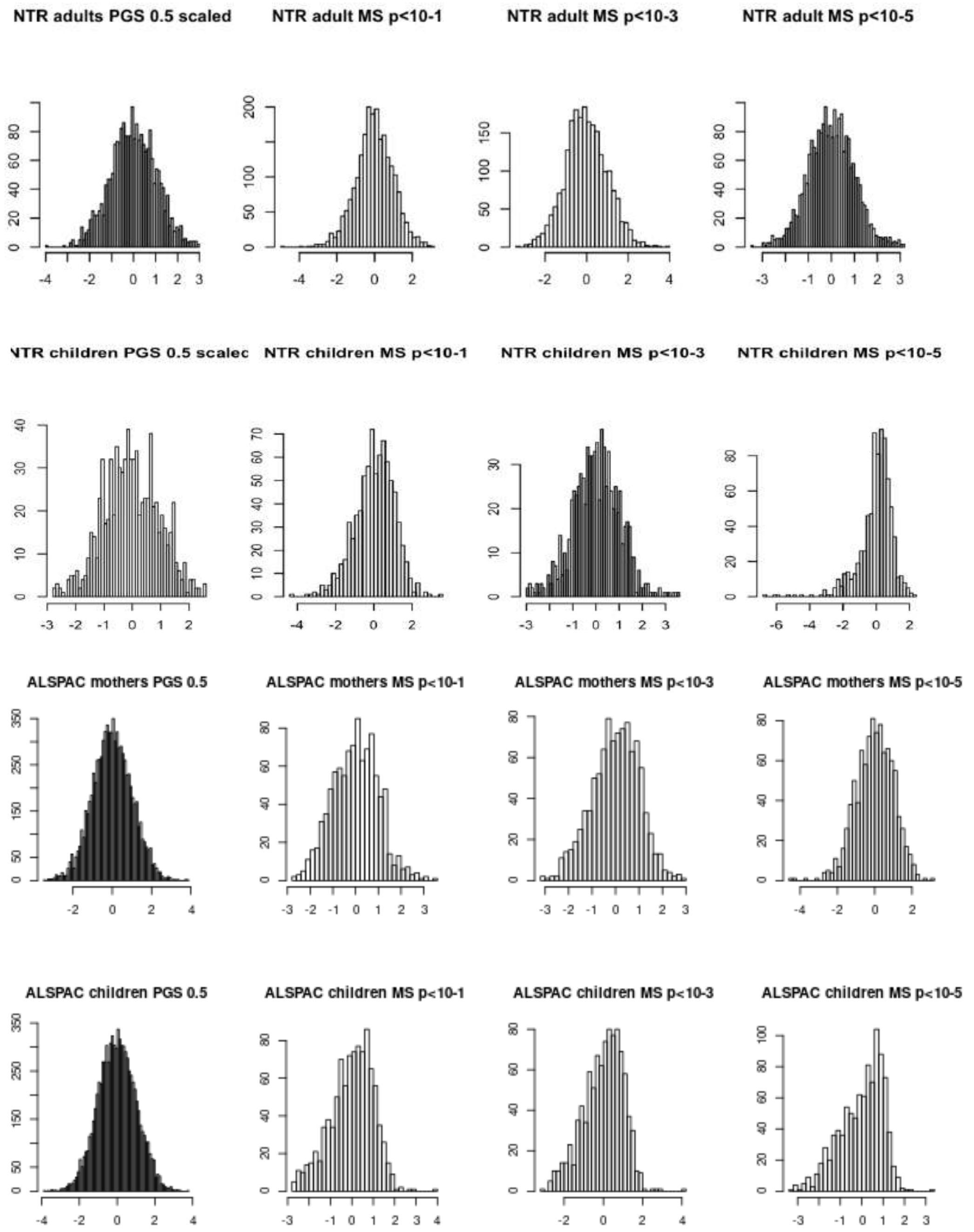

Supplementary Figure 14. Polygenic and methylation scores histograms
PGS, polygenic score; MS, methylation scores (with inclusion of CpGs at p<1x10^{-1}, p<1x10^{-3}, and p<1x10^{-5}).

Supplementary Table 31. Performance of left-handedness polygenic and methylation scores

	N	Model	score	ß	SE	*P*	R^2	R^2 (%)
**Same-age same tissue (whole blood)**								
NTR adults	2198	LH ~ PGS + GWAS covariates	PGS	0.0513	0.0632	0.4170	0.0008	0.0811
		LH ~ MS1 + PGS + GWAS covariates + EWAS covariates	PGS	0.0429	0.0650	0.5095	0.0005	0.0516
		LH ~ MS1 + PGS + GWAS covariates + EWAS covariates	MS $p<1x10^{-1}$	0.0094	0.0831	0.9101	0.00002	0.0017
		LH ~ MS2 + PGS + GWAS covariates + EWAS covariates	PGS	0.0409	0.0650	0.5294	0.0005	0.0473
		LH ~ MS2 + PGS + GWAS covariates + EWAS covariates	MS $p<1x10^{-3}$	-0.0447	0.0744	0.5479	0.0004	0.0422
		LH ~ MS3 + PGS + GWAS covariates + EWAS covariates	PGS	0.0407	0.0650	0.5317	0.0005	0.0453
		LH ~ MS3 + PGS + GWAS covariates + EWAS covariates	MS $p<1x10^{-5}$	-0.0984	0.0866	0.2561	0.0017	0.1701
ALSPAC mothers	574	LH ~ PGS + GWAS covariates	PGS	-0.178	0.162	0.272	0.00461	0.461
		LH ~ MS1 + PGS + GWAS covariates + EWAS covariates	PGS	-0.248	0.188	0.187	0.00372	0.372
		LH ~ MS1 + PGS + GWAS covariates + EWAS covariates	MS $p<1x10^{-1}$	0.00136	0.582	0.998	-0.00001	-0.0013
		LH ~ MS2 + PGS + GWAS covariates + EWAS covariates	PGS	-0.254	0.188	0.177	0.00399	0.399
		LH ~ MS2 + PGS + GWAS covariates + EWAS covariates	MS $p<1x10^{-3}$	-0.369	0.539	0.493	0.00383	0.383
		LH ~ MS3 + PGS + GWAS covariates + EWAS covariates	PGS	-0.234	0.189	0.216	0.00122	0.122
		LH ~ MS3 + PGS + GWAS covariates + EWAS covariates	MS $p<1x10^{-5}$	-0.183	0.228	0.423	-0.0017	-0.17
**Same-age different tissues (buccal cells and whole blood)**								
NTR at 9 years	799	LH ~ PGS + GWAS covariates	PGS	-0.016	0.103	0.877	0.00002	0.002
		LH ~ MS1 + PGS + GWAS covariates + EWAS covariates	PGS	0.007	0.108	0.950	0.00002	0.002
		LH ~ MS1 + PGS + GWAS covariates + EWAS covariates	MS $p<1x10^{-1}$	-0.047	0.129	0.715	0.00024	0.024
		LH ~ MS2 + PGS + GWAS covariates + EWAS covariates	PGS	0.013	0.108	0.902	0.00009	0.009
		LH ~ MS2 + PGS + GWAS covariates + EWAS covariates	MS $p<1x10^{-3}$	-0.126	0.107	0.236	0.00496	0.496
		LH ~ MS3 + PGS + GWAS covariates + EWAS covariates	PGS	0.005	0.108	0.960	0.00002	0.002
		LH ~ MS3 + PGS + GWAS LH + EWAS covariates	MS $p<1x10^{-5}$	-0.111	0.102	0.279	0.00218	0.218
ALSPAC at 7 years	630	LH ~ PGS + GWAS covariates	PGS	0.036	0.145	0.801	0.000	0.031
		LH ~ MS1 + PGS + GWAS covariates + EWAS covariates	PGS	0.116	0.157	0.460	0.003	0.348
		LH ~ MS1 + PGS + GWAS covariates + EWAS covariates	MS $p<1x10^{-1}$	0.030	1.070	0.978	0.000	0.000
		LH ~ MS2 + PGS + GWAS covariates + EWAS covariates	PGS	0.117	0.157	0.455	0.004	0.356
		LH ~ MS2 + PGS + GWAS covariates + EWAS covariates	MS $p<1x10^{-3}$	0.141	0.884	0.873	0.000	0.015
		LH ~ MS3 + PGS + GWAS covariates + EWAS covariates	PGS	0.122	0.157	0.438	0.004	0.414
		LH ~ MS3 + PGS + GWAS covariates + EWAS covariates	MS $p<1x10^{-5}$	0.677	0.415	0.103	0.013	1.282

LH, left-handedness (LH=1, RH=0); PGS, polygenic scores; MS, methylation scores with CpGs at three thresholds in EWAS (MS1 $p<1x10^{-1}$, MS2 $p<1x10^{-3}$, MS3 $p<1x10^{-5}$); R^2%, variance explained by score in percentages; *P*, *P*-value; α=0.05/4=0.0125
See calculation of explained variance in *Appendix 2*.

Supplementary Table 32. Meta-analysis summary statistics (basic model) (legend)

see SupplementaryTable_32.xlsx
Summary statistics from meta-analysis with basic model. cgid, Illumina probe ID. CHR, chromosome. Position, genome build Hg19 (build 37). Gene and Gene region, information on gene mapping to CpG. N_{NTR}, number of cases in NTR. N_{ALSPAC}, number of cases in ALSPAC. Weight, total number of cases in meta-analysis. β, regression coefficient. SE, standard error. q-value, false discovery rate value. Direction, direction of effect. N_{CpGs} = 409,563. N_{NTR} = 2710. N_{ALSPAC} = 1232.

Supplementary Table 33. Meta-analysis summary statistics (adjusted model) (legend)

see SupplementaryTable_33.xlsx
Summary statistics from meta-analysis with adjusted model. cgid, Illumina probe ID. CHR, chromosome. Position, genome build Hg19 (build 37). Gene and Gene region, information on gene mapping to CpG. N_{NTR}, number of cases in NTR. N_{ALSPAC}, number of cases in ALSPAC. Weight, total number of cases in meta-analysis. β, regression coefficient. SE, standard error. q-value, false discovery rate value. Direction, direction of effect. N_{CpGs} = 409,563. N_{NTR} = 2663. N_{ALSPAC} = 1058.
